# Feedback-controlled hydrogels with homeostatic oscillations and dissipative signal transduction

**DOI:** 10.1038/s41565-022-01241-x

**Published:** 2022-11-28

**Authors:** Hang Zhang, Hao Zeng, Amanda Eklund, Hongshuang Guo, Arri Priimagi, Olli Ikkala

**Affiliations:** 1 Department of Applied Physics, Aalto University, Espoo, Finland; 2 Smart Photonic Materials, Faculty of Engineering and Natural Sciences, Tampere University, Tampere, Finland

**Keywords:** Actuators, Sensors and biosensors

## Abstract

Driving systems out of equilibrium under feedback control is characteristic for living systems, where homeostasis and dissipative signal transduction facilitate complex responses. This feature not only inspires dissipative dynamic functionalities in synthetic systems but also poses great challenges in designing novel pathways. Here we report feedback-controlled systems comprising two coupled hydrogels driven by constant light, where the system can be tuned to undergo stable homeostatic self-oscillations or damped steady states of temperature. We demonstrate that stable temperature oscillations can be utilized for dynamic colours and cargo transport, whereas damped steady states enable signal transduction pathways. Here mechanical triggers cause temperature changes that lead to responses such as bending motions inspired by the single-touch mechanoresponse in *Mimosa pudica* and the frequency-gated snapping motion inspired by the plant arithmetic in the Venus flytrap. The proposed concepts suggest generalizable feedback pathways for dissipative dynamic materials and interactive soft robotics.

## Abstract (web summary)

An optical signal transduction pathway through feedback-controlled homeostatic temperature oscillations and mechanoresponse enables dynamic functionalities in a hydrogel.

## Main

Biological functionalities exploit dissipative coupled biochemical feedback loops across multiple length scales, providing, for example, homeostasis^1–4^, circadian rhythms^5^, adaptation^6^ and signal transduction^7–9^. They inspire dynamic synthetic materials^10,11^ beyond the classical biomimetic^12,13^ and stimulus-responsive materials^14^. In biological signal transduction, sensed stimuli are converted into transient intermediate signals, entrained through coupled feedback loops^5,15^, leading to responses^1,3,9,16^. Out-of-equilibrium conditions promote hopping over the potential barriers for biochemical reactions that ultimately fuel the responses^17^. In synthetic materials, despite the recent progresses in dissipative and feedback-controlled systems^18–22^ such as chemomechanical feedback^23,24^ and physical/chemical oscillators^25–29^, the implementation of homeostasis and signal transduction under out-of-equilibrium conditions remains underexplored. Implementing such synthetic systems would allow novel routes towards life-inspired functionalities for next-generation intelligent materials^10^.

**Fig. 1:**
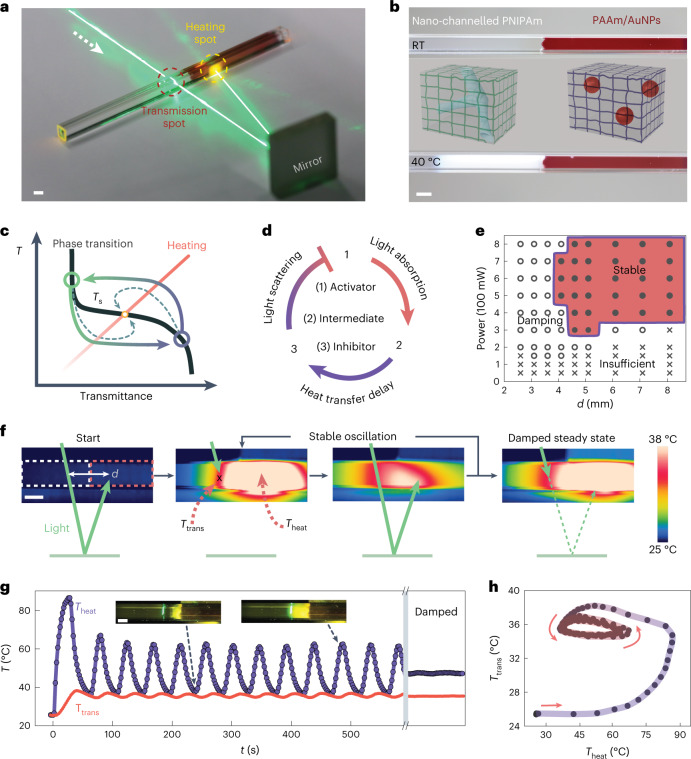
Light-driven nano-functionalized hydrogel system showing temperature oscillation based on a negative feedback loop with controlled time delay. **a**, Illustration of the experimental setup. **b**, Visual appearance of the gels encapsulated in a glass capillary at room temperature (RT) (top) and 40 °C (bottom). **c**, Schematic of the interplay between temperature and photothermal heating of PAAm/AuNP as a function of the transmittance of the thermoresponsive nano-channelled PNIPAm. The arrowed loop indicates stable oscillations with delay. The dashed lines indicate damped oscillation, stabilizing at the steady-state temperature *T*_s_. **d**, Illustration of the negative feedback loop with a delay between photothermal heating and the resultant light scattering from phase transition of the channelled PNIPAm hydrogel. **e**, Stability map of oscillation depending on the power and delay distance *d*. ‘Stable’ indicates oscillation longer than 20 min. ‘Insufficient’ power leads to temperatures lower than LCST at the transmission spot. **f**, Schematic of the feedback cycles and corresponding infrared images with dashed frames marking the contour of the two gels (white, nano-channelled PNIPAm; red, PAAm/AuNPs). Temperatures of the heating (*T*_heat_) and transmission (*T*_trans_) spots and distance *d* are indicated. **g**, Stable oscillation of *T*_heat_ and *T*_trans_ under constant irradiation shown for 600 s, as well as an example of the damped steady state. **h**, *T*_heat_ versus *T*_trans_ from **g** showing the stable limit cycle of oscillation. Light, 600 mW, 532 nm; *d* = 4.6 mm. Scale bars, 2 mm. Source data

**Extended Data Fig. 1:**
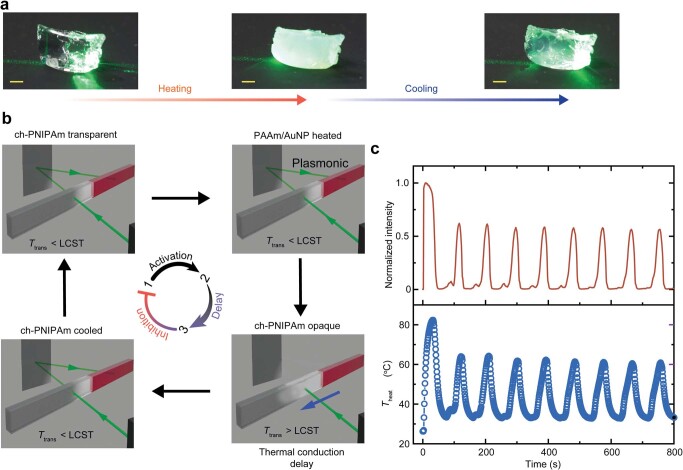
Light driven self-oscillation in thermally coupled gels. **a**, Photographs of nano-channelled PNIPAm gel under 532 nm laser irradiation, when heated above LCST (by a heat gun) and cooled down to room temperature. Scale bars: 2 mm. **b**, Schematic illustration of light propagation during one oscillation cycle. **c**, Normalized light intensity in transmission and the *T*_heat_ during the oscillation cycles. Power: 800 mW; delay distance: 5.7 mm. Source data

Here we report a system consisting of two interfaced nano-functionalized hydrogels, which are covalently encapsulated in a glass tube to prevent undesired swelling and shrinking, fuelled by a constant laser beam (Fig. 1a and Extended Data Fig. 1). The beam passes through a thermoresponsive gel and is impinged via a mirror onto a light-absorbing gel. Under a time delay, the photothermal heating of the absorbing gel causes the temperature rise of the thermoresponsive gel, inducing fast phase transition and blocking the light transmittance. The delay in this feedback process is controlled by thermal conduction across the distance between the transmission and heating spots. By tuning the feedback loop, we achieve either robust stable oscillations driving dissipative functionalities or damped steady states of temperature, whose transient overshoot allows artificial signal transduction on coupling with other thermoresponsive systems.

## Light-fuelled tunable self-oscillation via negative feedback

The thermoresponsive gel consists of nano-channelled poly(*N*-isopropylacrylamide) (PNIPAm) where interconnected porous channels are formed by removing a sacrificial template. This allows sufficient heating-induced opaqueness above the lower critical solution temperature (LCST) at *T*_LCST_ ≈ 36 °C and facilitates rapid thermal transitions (Fig. 1b, Supplementary Fig. 1 and Methods)^30^. At *T* < *T*_LCST_, a constant laser beam at 532 nm passes through the initially transparent channelled PNIPAm at the transmission spot (temperature *T*_trans_) and is subsequently reflected to polyacrylamide (PAAm) gel via a mirror, where the light is absorbed at the heating spot (temperature *T*_heat_) by physically trapped gold nanoparticles (AuNPs) (Fig. 1a,b and Supplementary Figs. 2 and 3). This leads to local photothermal heating at the laser spot, which is transferred to the channelled PNIPAm via thermal conduction with time delay Δ*t* determined by distance *d* between the heating and transmission spots. Once *T*_trans_ increases above *T*_LCST_, the incident beam is blocked due to optical scattering, which prevents the beam from reaching the PAAm/AuNP side. As a result, the whole system cools down. Once *T*_trans_ falls below *T*_LCST_, the beam is allowed to pass again, and a new heating–cooling cycle starts. The photothermally induced temperature increase is directly proportional to light intensity^31^, whereas transmittance at the transmission spot follows the typical LCST curve of PNIPAm (Fig. 1c and Supplementary Fig. 1)^30^. Here large nonlinearity in the phase transition of nano-channelled PNIPAm and sufficiently large delay in heat transfer prevent the gel from settling on a steady state at the intersect *T*_s_. If no delay is present or if the transmission spot size is too large, no oscillation is observed (Supplementary Figs. 4 and 5). Besides, stable and regular oscillations cannot be achieved with conventional PNIPAm hydrogel due to the slow response and broad temperature range of phase transition (Supplementary Fig. 6), thus underpinning the importance of nanochannels.

In a broader perspective, biochemical oscillators are typically based on negative feedback loops with a delay consisting of the (1) activator, (2) intermediate and (3) inhibitor^5^. In our system (Fig. 1d), the constant light beam serves as the activator, the resultant photothermal heating of AuNPs in PAAm gel acts as the intermediate and thermally switchable phase transition of channelled PNIPAm gel is the inhibitor.

**Extended Data Fig. 2:**
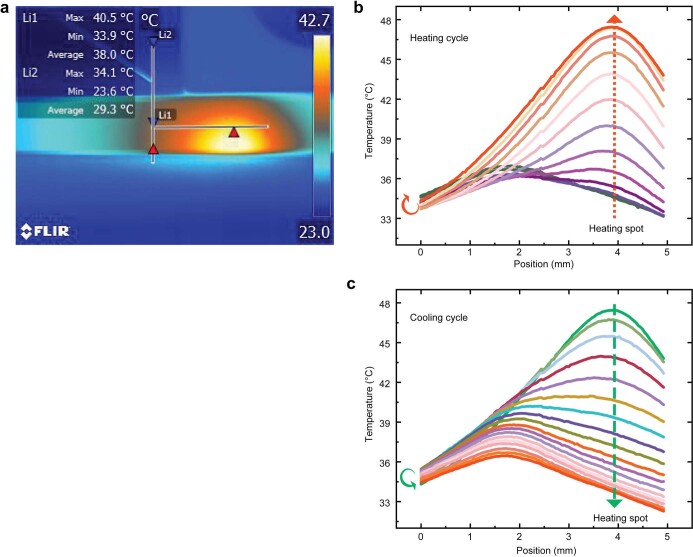
Temperature evolution during one oscillation cycle. **a**, Thermal camera image of the capillary. **b**, Temperature along the centreline (Li1) during the heating cycle. **c**, Temperature along the centreline during the cooling cycle. The centre of the heating spot is marked by the dashed arrows. The transmission spot is located at position 0 mm. Laser power: 600 mW. Source data

The two key parameters determining the oscillation stability are laser power *P* and delay distance *d*. Figure 1e suggests that a minimum *P* value of 300 mW is required to trigger the phase transition and initiate the temperature oscillations. For insufficient *P* (<150 mW at most distances), *T*_trans_ cannot reach the LCST to provide the negative feedback (Supplementary Fig. 7). On the other hand, too high power (≥900 mW) leads to the boiling of water, causing failure of the gel. Large delays sustain stable (>1 h) self-oscillations, whereas for short delays, damping of oscillation takes place more easily (Supplementary Figs. 7–9). The temperature evolution during the oscillation, damping and limit cycle are illustrated in Fig. 1f–h and Supplementary Videos 1 and 2. At the onset of irradiation, there exists an initial temperature overshoot that stabilizes after few oscillation cycles, whereas the damped state yields steady temperatures. The temperature profiles of the whole gel during the heating and cooling cycles are shown in Extended Data Fig. 2.

**Fig. 2:**
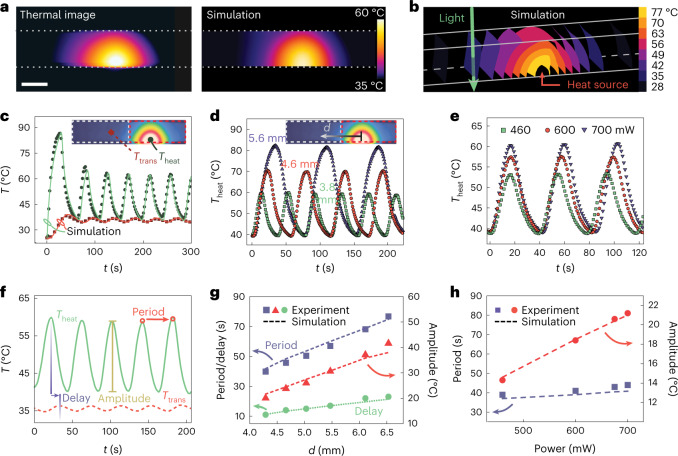
Control of temperature oscillation: experiments and simulations. **a**, Thermal image of the oscillator and the corresponding simulation. The dotted lines indicate the walls of the glass capillary. Scale bar, 2 mm. **b**, Simulated three-dimensional isothermal surfaces inside the oscillator. The white lines indicate the walls of the capillary. The green arrow indicates the laser beam at the transmission spot. **c**, Oscillation data from Fig. 1g (solid dots and squares), together with the simulation result (curves). **d**, Distance-dependent oscillation at constant power (600 mW) normalized at *t* = 0 s. **e**, Power-dependent oscillation at fixed *d* (4.1 mm) normalized at *t* = 0 s. **f**, Illustration of the delay, period and amplitude of the oscillation. **g**, Distance dependence of period, delay and amplitude at the heating spot at constant power (600 mW). The dashed lines interpolate the simulated results at the corresponding experimental data points. **h**, Power dependence of the period and amplitude at the heating spot at a fixed distance (4.1 mm). The dashed lines interpolate the simulated results at the corresponding experimental data points. Source data

Figure 2a shows the experimental thermal image at the heating spot, revealing the thermal gradient, compared with finite element modelling (Supplementary Fig. 10 and Methods). When moving away from the heating spot, the curvature of the isothermal surface decreases (Fig. 2b and Supplementary Video 3). Despite the simplifications, the simulation is consistent with the experimental data (Fig. 2c). The periodicity and amplitude of the oscillation as a function of power *P* and distance *d* are quantified in Fig. 2d,e, where *d*, in particular, has a strong influence on the oscillation characteristics (period, amplitude and delay; Fig. 2f). A linear dependence on *d* can be found for all these parameters, both experimentally and numerically (Fig. 2g). This is because the phase transition in channelled PNIPAm (timescale at tens of milliseconds)^30^ is considerably faster than the heat transfer process through the gel tube (tens of seconds). Therefore, the oscillation periodicity is predominantly controlled by the timescale of heat transfer, which is linearly dependent on *d* (Supplementary Fig. 11). Figure 2g shows that the period (from 40 to 77 s) and amplitude (from 20 to 41 °C) double as *d* increases from 4.3 to 6.5 mm, which can be attributed to the higher time delay and temperature gradient between the heating and transmission spots at longer *d*. In contrast, the laser power has only a minor influence on periodicity but strongly affects the amplitude (Fig. 2h). For the correlations among power, distance, amplitude and periodicity, the experimental and simulated data are provided in Supplementary Figs. 12–15. It should be noted that the light power used in our setup is comparable to other photothermally driven hydrogel systems^32,33^, whereas numerical simulation suggests that the power may be reduced by one order of magnitude by scaling down the device dimensions by a factor of four (Supplementary Fig. 16).

## Robust and homeostatic self-oscillation

**Fig. 3:**
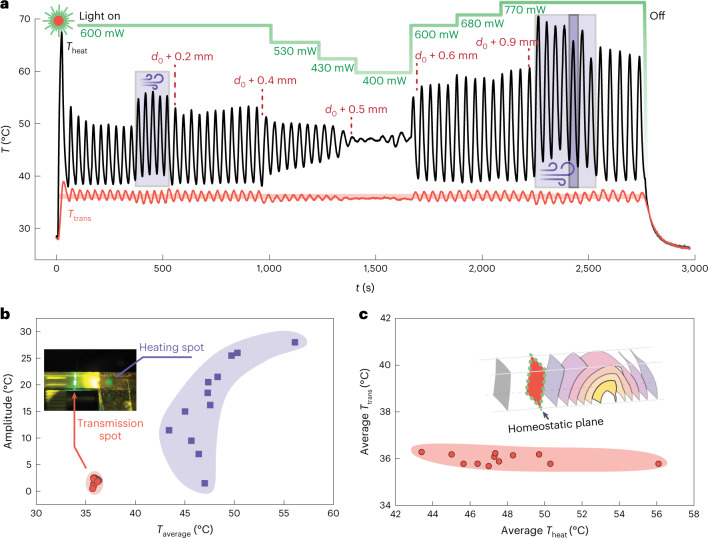
Robust homeostatic oscillation. **a**, Temperature oscillations under different disturbances and changing conditions (cooling by air blowing, delay *d* and laser power change). The green line indicates the change in laser intensity; the purple shades indicate air flow; the change in *d* is indicated by the red dashed lines. Here *d*_0_ = 3.7 mm. **b**, Time-averaged temperature versus amplitude of oscillation at the transmission and heating spots. **c**, Time-averaged temperature at *T*_trans_ versus that at *T*_heat_. Source data

Inspired by the stability of biological homeostasis against disturbances, which typically involves negative feedback loops, we next show that the present gel oscillator is also robust against external disturbances or changing conditions. Figure 3a shows the influence of cooling by forced air flow, changing delay distance and changing incident light power on the self-sustained oscillation. The air flow mimics one of the most common environmental change, that is, wind, which may cause shivering in homeothermic species to increase the heat production. Interestingly, even a weak air flow of 0.3 ± 0.1 m s^−1^ (0 on the Beaufort scale) leads to an increased mean value and amplitude of *T*_heat_, indicating autonomous adjustment to stronger photothermal heating that stabilizes the *T*_trans_ value around the LCST. On such air flow, *T*_trans_ immediately drops by about 0.4 °C, which is sufficient to cause a transient reduction in opaqueness that enhances light transmittance at the transmission spot. Consequently, *T*_heat_ increases to compensate for the additional heat loss such that *T*_trans_ returns to its homeostatic point and reduces the light transmission. Thus, the whole system is controlled by the negative feedback mediated by the change in gel opaqueness at the transmission spot. The oscillation is also robust to a strong air flow (1 m s^−1^ and 2,420 s; Fig. 3a), showing full recovery after the air flow ceases.

**Extended Data Fig. 3:**
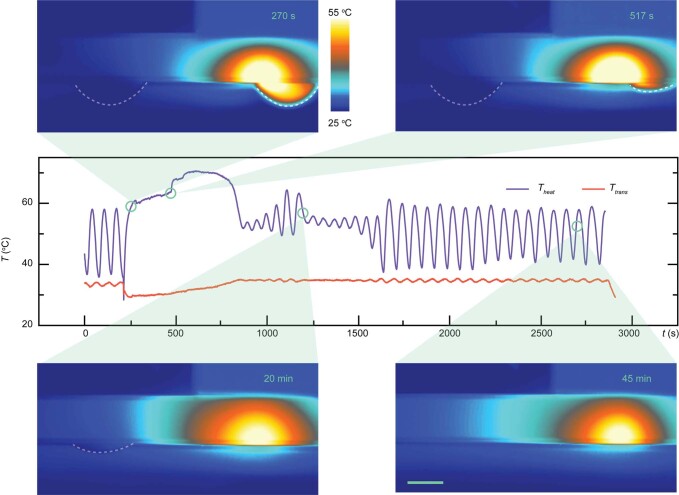
Dormancy of temperature oscillation by addition of water droplets. Oscillation upon addition of a water droplet at 214 s. Dashed frames in the thermal camera images show the droplets. Once the droplets are evaporated (1600 s), the oscillation fully recovers. Laser power: 600 mW; Delay distance: 5.9 mm; Scale bar: 2 mm. Source data

Both incident light power and *d* have a strong influence on the oscillation amplitude of *T*_heat_. This is evident in Fig. 3a: by increasing the laser power from 400 to 770 mW and *d* by 0.4 mm, the amplitude of *T*_heat_ increases by 20 °C. In contrast, the amplitude of *T*_trans_ only varies by 1.8 °C. The temperature stability of the two spots under external disturbances or changing conditions is summarized in Fig. 3b,c, evidencing that although the amplitude and average value of *T*_heat_ undergo large variations, *T*_trans_ remains strikingly stable at around 36 °C, similar to biological homeostasis. As is the case in biological systems, excessive disturbances such as a strong change in heat dissipation may temporarily drive the system out of the steady state, but the system recovers once the disturbances are removed. For instance, adding water droplets on the gel tube leads to a transient dormancy of oscillation until the water has evaporated (Extended Data Fig. 3).

## Dissipative functionalities via stable self-oscillation

**Fig. 4:**
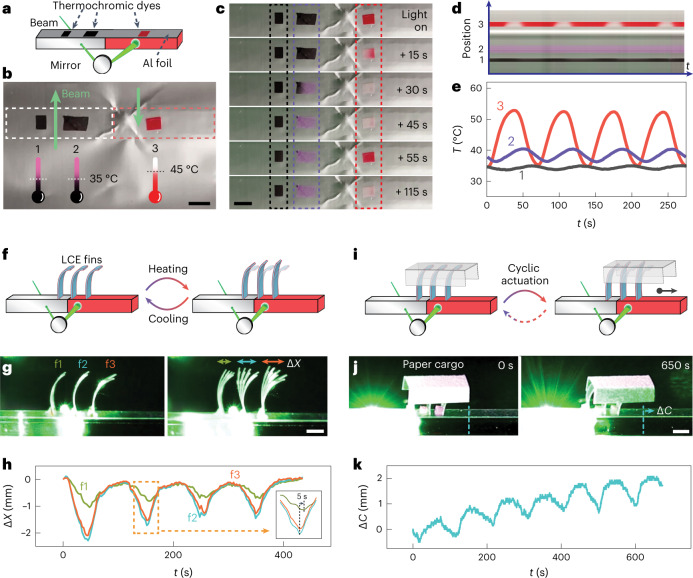
Dissipative colour display and sustained cargo transport based on stable oscillations. **a**, Illustration of thermochromic stickers on top of the gel tube covered by an aluminium foil. **b**, Photograph of the stickers at room temperature with the corresponding schematic showing thermochromic colours and transition temperatures. The laser beam positions are indicated by the green arrows. The dashed frames mark the contours of the two gels (white, nano-channelled PNIPAm; red, PAAm/AuNPs). **c**, Photograph series of the colour change at the onset of irradiation. **d**, Kymograph of the stickers under stable oscillations. **e**, Temperature oscillations at spots corresponding to the positions marked in **b** and **d**. Light power, 700 mW; *d* = 6.1 mm. **f**, Illustration of a gel–LCE assembly consisting of an array of LCE fins attached to the gel oscillator that undergo synchronized but non-identical deformations under temperature oscillations. **g**, Photograph of the LCE fins (left) and the superimposed image of the fins during one cycle of deformation (right). **h**, Horizontal displacement (Δ*X*) of the fin tips corresponding to **g**. The inset shows the zoomed-in view of one cycle showing the time lag between f1 and f2. **i**, Illustration of a paper cargo being transported by the deformation of LCE fins. **j**, Photographs of the paper cargo before (left) and after (right) light-driven cyclic actuations. **k**, Horizontal displacement of the centre of mass of the cargo with time. Light power, 700 mW; *d* = 8.1 mm. Scale bars, 2 mm. Source data

Next, we demonstrate that stable self-oscillations can be used to drive responsive materials for obtaining dissipative functionalities. As the first example (Fig. 4a–e and Supplementary Video 4), the light-fuelled thermal self-oscillation is coupled with thermochromic dyes to achieve dynamic colour displays. Two types of dye are used: one that transitions from black to pink on heating above 35 °C and the other from red to white at 45 °C (Fig. 4b). The dyes are mixed with polydimethylsiloxane to form solid stickers placed on top of the gel tube covered by an aluminium foil, which is used to block scattered laser light from reaching the stickers. Under stable oscillations, the stickers’ temperature and thus the colour strongly depends on the position: the further away from the heating spot, the lower the temperature and oscillation amplitude (Extended Data Fig. 2 and Supplementary Fig. 11). Three colour modes under dissipative conditions are achieved (Fig. 4c–e). At position 3, that is, close to the heating spot, a blinking pattern between red and white colours is observed with a periodicity corresponding to the temperature oscillations. Position 2 produces a sustained display of pink colour as a result of elevated temperature above 35 °C, whereas further away from the transmission spot at position 1, a steady appearance of black colour ensues from the local temperature below 35 °C (Fig. 4d,e). The conversion of temperature oscillation into dynamic colours demonstrates the potential for applications such as visual signalling and sensing.

On the other hand, periodic waving motion driven by constant light can be achieved by attaching an array of thermoresponsive liquid crystal elastomer (LCE) fins to the tube (Fig. 4f–h). The splay-aligned LCE fins undergo thermally driven reversible bending due to asymmetric thermal expansion (Supplementary Fig. 17)^34,35^. Cyclic temperature oscillations drive the fins to wave (Fig. 4g, right). The deformations of fins are synchronized with temperature oscillations, whereas they adopt non-identical magnitude and a minor time delay of few seconds due to the difference in their positions relative to the heating spot (Fig. 4h and Supplementary Fig. 11). This asymmetry in deformation, time delay between fins and tilted orientation of the fins enable friction bias on a model cargo made of paper (Fig. 4i–k and Supplementary Video 5), which undergoes horizontal displacement driven by cyclic oscillations. Even though the net translational speed is relatively slow (200 μm min^−1^), this demonstration reveals the possibility of constructing autonomous active transport systems under feedback control, using a non-modulated light source.

## Mechanoresponsive signal transduction via damped steady states

**Fig. 5:**
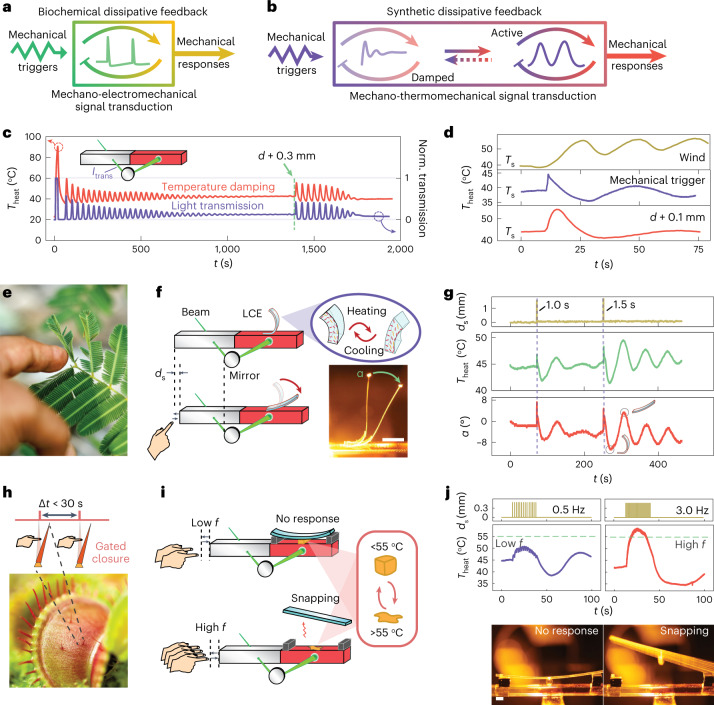
Feedback-controlled artificial dissipative signal transduction of mechanical stimuli. **a**, Schematic of the mechano-electromechanical signal transduction in plants based on action potential intermediates. **b**, Schematic of the artificial mechanoresponsive signal transduction with transient temperature intermediates. **c**, Damped oscillation and its revival induced by a mechanical trigger. Power, 700 mW; *d* = 3.8 mm. Normalized transmission is the light power (*I*_trans_) through the channelled PNIPAm gel normalized to the initial light power below LCST (*I*_0_). **d**, Examples of different stimuli to excite the transient thermal variation from a damped state. A mechanical trigger causes a transient horizontal shift (0.3 mm for 1 s) of the gel relative to the laser. **e**, Photograph of *M. pudica* showing mechanosensitive actuation by a single mechanical stimulus. **f**, Illustration of mechanoresponsive system based on a gel–LCE assembly, where the LCE undergoes reversible thermal actuation in response to mechanical stimulus. **g**, Plot of gel temperature variation and the LCE strip bending angle *α* under transient mechanical stimuli. **h**, Photograph of a Venus flytrap with its sensory hairs counting the trigger interval for the closure of the trap. **i**, Illustration of frequency-gated irreversible mechanoresponsive system based on the gel–snapper assembly, consisting of a pre-stressed plastic film and a temperature-sensitive glue with a solid-to-liquid transition at 55 °C. **j**, Temperature elevation of the gel in the snapper assembly on low- and high-frequency mechanical triggering (top). Photographs of the snapper assembly, showing no response on low-frequency triggering and snapping on high-frequency triggering (bottom). Scale bars, 2 mm. Light power, 700 mW. Source data

In a general perspective, driving systems out of equilibrium can allow promoted stimulus responses^17^ in ways not achievable in conventional equilibrium material systems^14,36^. We will next demonstrate two mechanoresponsive systems, inspired by biological functions^37^ and signal transduction via action potential in plants (Fig. 5a,b)^8,38^. A classical mechanoresponsive model system is *Mimosa pudica* where a single touch leads to rapid closure (Fig. 5e)^8^ of the leaf, whereas a Venus flytrap (*Dionaea muscipula*) has a more subtle response by counting the number and time interval of mechanical touches^38^. In our system, the gel enters feedback-controlled damped steady states after certain duration of oscillation, most notably at small *d*, in which the temperature oscillation can be revived on the application of an external mechanical trigger (Fig. 5c). Such a damped state is highly sensitive to the type and magnitude of applied stimuli (Fig. 5d and Supplementary Figs. 18–21), which can be exploited for constructing dissipative signal transduction pathways under out-of-equilibrium conditions.

We first study single-touch mechanical responses by attaching an LCE strip to the gel tube (Fig. 5f). The strip stays still when the oscillator is in the damped steady state. On horizontal displacement (~1.5 mm) of the gel tube relative to the laser beam, the light beam impinging on the channelled PNIPAm shifts to an area of higher transparency. This leads to an instantaneous increase in the transmitted light intensity at the transmission spot and subsequent thermal overshoot at the heating spot, giving rise to thermally induced bending of the LCE (Fig. 5f and Supplementary Fig. 21). Once the trigger is removed, the curvature of the LCE recovers after a few periods of oscillation. The correlation between *T*_heat_ and change in bending angle of the LCE (Δ*α*) is exemplified in Fig. 5g, whereas the reversibility of the triggering process is shown in Supplementary Video 6. Supplementary Fig. 21 shows that the rise in *T*_heat_ on mechanical triggering is caused by increased light transmission. Subsequently, the extra energy input at the heating spot is conducted to the transmission spot to elevate *T*_trans_ after cessation of the trigger. This further reduces the gel transparency and thus *T*_heat_ to even below the damped steady-state temperature, causing few cycles of oscillations before the temperature is fully damped again. The evolution of *T*_heat_, light transmission and *T*_trans_ on a single trigger is provided in Supplementary Fig. 21b. Due to this feedback process, a transient mechanical trigger (<2 s) of the gel position is converted to a much longer temperature/actuator oscillation cycle of about 3 min (Fig. 5g). Thus, the dissipative mechanoresponsive actuator is based on the disturbance of the optothermal feedback loop. Besides, the response is powered by the energy input (light) of the feedback loop instead of the external trigger, in contrast to conventional stimuli-responsive materials.

We next show frequency-gated mechanoresponses algorithmically inspired by the Venus flytrap (Fig. 5h), which closes when there are two consecutive mechanical triggers on the hair taking place within 30 s, thus signifying the presence of a moving insect and preventing false triggering^38^. Although biological systems use electric pulses for generating responses, our synthetic system is based on analogue signals in temperature oscillations. In the steady state, *T*_heat_ only increases by 1 °C on the low-frequency (0.25 Hz) mechanical triggering of the gel. In contrast, on 2 Hz triggering, more than 5 °C increase is observed (Supplementary Fig. 22). The principle is based on the same effect described above, where mechanical triggers temporarily shift the laser beam at the transmission spot towards higher transparency, causing an additional heating effect. Since the duration of each trigger is almost the same, a higher frequency leads to a higher time-averaged light power input through the transmission spot. This can be observed by the positive correlations between the frequency and *T*_heat_ (Supplementary Fig. 22a,b). On the other hand, the maximum increase in *T*_heat_ is controlled by the feedback loop: once the additional heat propagates to the transmission spot, the transparency decreases for reducing the heating power (Supplementary Fig. 22c). This sets the upper limit of *T*_heat_, even if the trigger continues (Supplementary Figs. 22a,b and 23a). In this way, the maximum *T*_heat_ caused by periodic triggers is determined by the frequency of triggers, which can be further tuned by the laser power and *d* (Supplementary Fig. 23).

**Extended Data Fig. 4:**
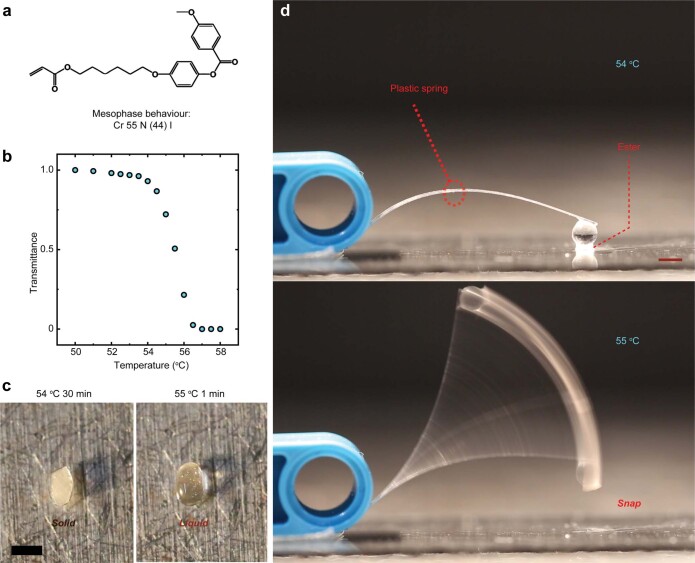
Temperature-gated snapping. The temperature threshold (55 °C) is determined by a thermoresponsive glue that suddenly releases the elastic energy from a prestressed spring. **a**, Chemical structure of the liquid crystal ester glue used for thermally induced release. **b**, Phase transition (crystal-to-liquid) behaviour of the glue, measured by cross polarized light transmission. **c**, Heat-induced melting of the glue (melting point 55 °C). **d**, Photographs of the heat-induced melting, yielding snapping of the plastic spring. Scale bars: 2 mm. Source data

**Extended Data Fig. 5:**
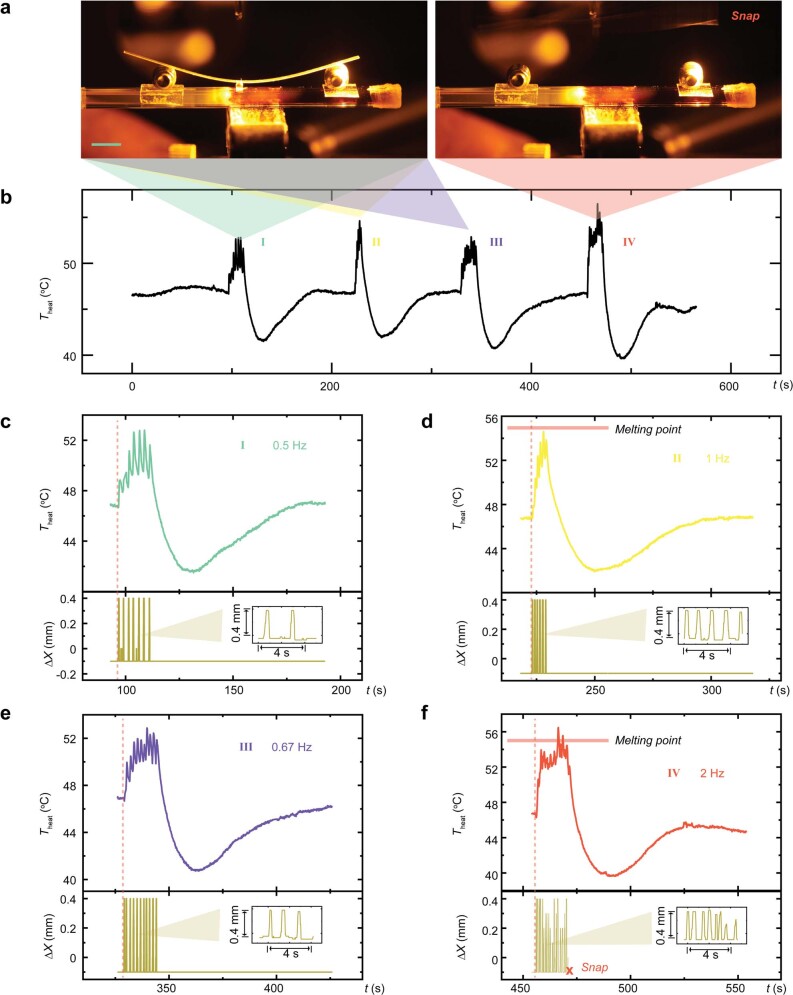
Frequency-gated snapping based on prestressed elastic sample and a thermoresponsive glue. **a**, Photographs of the gel-snapper assembly, showing no response upon low-frequency mechanical triggering and snapping upon high-frequency triggering, due to melting of the thermoresponsive glue. Scale bar is 5 mm. **b**, Change of temperature during the mechanical triggering. Zoom-in data of temperature change upon 0.5 Hz (**c**), 1 Hz (**d**), 0.67 Hz (**e**) and 2 Hz (**f**) triggering and the related sample position change Δ*X*. Light power: 700 mW. Insets in (**c**-**f**) are zoom-in data of Δ*X* change upon repeated triggers. Source data

To realize a frequency-gated device via the signal transduction pathway, we constructed a model system of mechanoresponsive snapper (Fig. 5i and Extended Data Fig. 4), which consists of a pre-stressed elastic cantilever attached to the heating spot of the gel by a thermosensitive glue^39^ (Extended Data Fig. 4a). The design principle is based on the melting of glue at an elevated temperature induced by high-frequency mechanical triggers, which causes the release and snapping motion of the cantilever. To initiate snapping, the condition Δ*T*_heat_ > *T*_m_ – *T*_s_ must be satisfied, where Δ*T*_heat_ is the temperature increase from the damped state, *T*_m_ is the melting temperature of the glue and *T*_s_ is the damped steady-state temperature. Since Δ*T*_heat_ increases with frequency, *T*_m_ can be readily tailored by the chemical composition (Supplementary Fig. 24), and *T*_s_ can be tuned by a change in *P* and *d* (Supplementary Fig. 25), one can program the system to allow frequency-gated snapping events under different conditions. As an illustrative example, Fig. 5i,j shows the irreversible snapping response only on high-frequency triggers, where a glue with *T*_m_ of 55 °C was used (Fig. 5j and Supplementary Video 7). Details on the temperature evolution on triggering at different frequencies are shown in Extended Data Fig. 5 and Supplementary Fig. 26.

The above signal transduction pathway can be generalized from the mechanical trigger to other stimuli. As shown in Fig. 5d and Supplementary Figs. 18–20, blowing wind and shift in *d* restores the transient oscillation behaviour or triggers transient thermal variations that can be implemented in the transduction scheme.

## Conclusions

We have realized life-inspired dissipative systems possessing robust homeostatic oscillations and dissipative mechanical signal transductions. Both functionalities are based on a light-driven negative feedback loop with thermally coupled delay, achieved in an encapsulated hydrogel system comprising thermoresponsive nano-channelled PNIPAm and light-absorbing PAAm gels containing AuNPs. We have shown stable temperature oscillations in the range of ±1 °C independent of laser power, delay distance, air flow or mechanical disturbances, where the amplitude and periodicity can be tuned by the input power and thermoresponsive delay. By leveraging stable temperature oscillations, we demonstrate a dynamic colour display and cargo transport systems. On the other hand, the system in the damped steady states exhibits sensitivity to the type, amplitude and frequency of disturbances, which can be utilized to construct transduction pathways. A gel–LCE assembly senses mechanical triggers and performs waving motion, algorithmically resembling the transient mechanosensing behaviour of *M. pudica*. Furthermore, a gel–snapper assembly is programmed to show frequency-gated mechanoresponses, as inspired by the number-counting mechanoresponse of the Venus flytrap. Although the demonstrated artificial signal transduction is highly simplified compared with their natural counterparts, they showcase dissipative functionalities that have not yet been demonstrated in conventional stimuli-responsive systems. The life-inspired concepts in the present model system suggest generalizable transduction pathways for dissipative dynamic materials and interactive soft robotics.

## Methods

### Materials

*N*‐Isopropylacrylamide (NIPAm, 99.0%, recrystallized from *n*‐hexane), poly(ethylene glycol) diacrylate (PEGDA, *M*_n_ = 10,000), *N*,*N*′‐methylenebisacrylamide (BIS, ≥99.5%), acrylamide (AAm, ≥99.0%), agarose (ultralow gelling temperature, A5030), gold(iii) chloride trihydrate (HAuCl_4_·3H_2_O, >99.9%), sodium citrate tribasic dihydrate (BioUltra, 99.5%), poly(ethylene glycol) methyl ether thiol (PEG‐SH, *M*_n_ = 2,000), 3‐(trimethoxysilyl)propyl acrylate (≥92.0%), 2‐hydroxy‐4′‐(2‐hydroxyethoxy)‐2‐methylpropiophenone (photoinitiator, Irgacure 2959, 98.0%) and 2,2-dimethoxy-2-phenylacetophenone were purchased from Sigma‐Aldrich. 1,4-Bis-[4-(6-acryloyloxyhexyloxy)benzoyloxy]-2-methylbenzene was purchased from SYNTHON Chemicals. 6-Amino-1-hexanol and dodecylamine were purchased from TCI. Sodium hydroxide (97%) and hydrochloric acid (1 M) were purchased from Fisher Scientific. Ethanol (99.5%) was purchased from Altia Oyj. Deionized water (18.2 MΩ; Direct-Q 3 ultraviolet (UV), Millipore) was used in all the experiments.

### Synthesis and modification of AuNPs

Citrate‐stabilized AuNPs were prepared by the classical citrate reduction of gold salt in water^40^. Briefly, trisodium citrate solution (2 ml, 1.00 wt%) was quickly injected in a boiling aqueous solution of HAuCl_4_·3H_2_O (100 ml, 0.01 wt%) under vigorous stirring. The solution was further refluxed for 10 min under stirring to complete the reaction. The AuNPs were analysed by transmission electron microscopy (Tecnai 12), which showed an average diameter of 20.0 ± 2.3 nm (Supplementary Fig. 2). The 20 nm AuNPs were stabilized using PEG‐SH by adding an ethanolic PEG‐SH solution (8 ml, 5 mg ml^−1^) to the AuNP solution (85 ml), which was incubated overnight on an orbital shaker. Finally, the modified AuNPs were purified three times by centrifugation (16,000×*g* for 25 min) and rediluted in pure water to yield a concentrated stock solution (0.88 ml) of PEGylated AuNPs. The concentration of AuNPs in this stock solution has been measured to be 4.3 mg ml^−1^ according to the dry weight, which is slightly lower than the theoretical value of 4.8 mg ml^−1^ due to loss during centrifugation. The concentration of AuNPs in the PAAm gel is, thus, 0.86 mg ml^−1^.

### Preparation of gel oscillators

To prevent the undesired swelling and shrinking of hydrogels, the capillaries have been silanized to introduce covalent bonds with the hydrogels. Borosilicate glass tubes with a square cross section (2.0 mm × 2.0 mm (inner) and 2.8 mm × 2.8 mm (outer), VitroTubes) were cut into an appropriate length (~5 cm) with a glass cutter and cleaned by sonication in deionized water. The tubes were then activated by oxygen plasma for 5 min (Pico, Diener Electronic) and functionalized by storing the slides overnight in an evacuated desiccator containing 100 µl of 3‐(trimethoxysilyl)propyl methacrylate at 1 × 10^−1^ mbar. Subsequently, the liquid silane was removed, and the desiccator was further evacuated to 1 × 10^−3^ mbar for 2 h to remove any unbound silane on the glass surface. The silanized glass slides were stored in a sealed vial in the fridge and used within a week after preparation.

To prepare the gel, the desired amount of agarose was dissolved in deionized water by heating and vortexing until full dissolution to make a 1 wt% stock solution. Then, 0.25 ml of the hot agarose solution was added together with 0.20 ml water to dissolve 50.0 mg NIPAm, 1.0 mg photoinitiator Irgacure 2959 and 4.4 mg PEGDA crosslinker. The resulting solution, thus, contained 10.0 wt% NIPAm, 0.5 wt% agarose and 0.1 mol% PEGDA relative to NIPAm. The thoroughly mixed solution was then degassed by nitrogen bubbling for 5 min in a 40 °C water bath to prevent the gelation of agarose. The degassed solution was transferred to fill half of a silanized glass tube, which was sealed with Parafilm and kept in a nitrogenated vial. The tube was then stored in a fridge at 4 °C for 30 min for the gelation of agarose and then irradiated in a UV reactor (8 × 14 W lamps, 350 nm, Rayonet) for 20 min for the polymerization of NIPAm. Afterwards, the glass tube was filled with a degassed aqueous solution containing 10 wt% AAm, 1 mol% BIS and 1 mol% photoinitiator relative to AAm and AuNPs. The concentration of AuNPs corresponds to an optical density of 2 at 532 nm for an optical path of 2 mm (Supplementary Fig. 2), calibrated by a UV–visible spectrometer (Cary 5000, Agilent). Finally, the polymerization of AAm gel was carried out in a UV reactor (8 × 14 W lamps, 350 nm, Rayonet) for 20 min. The resulting hydrogel in the tube was purified by incubation in a water bath at 60 °C for 30 min and then kept overnight in pure water at room temperature. In this way, the agarose network was removed to form channelled PNIPAm. The tubes were stored in deionized water before use. Oscillators with other compositions were prepared following the same protocol.

### Simulation of oscillator

The simulation of the oscillator was carried out in COMSOL Multiphysics 5.5 using a heat transfer module. The geometry parameters used to set up the three-dimensional model are summarized in Supplementary Table 1 and Supplementary Fig. 10. It is assumed that the two gels possess the same physical parameters (heat capacity, thermal conductivity and density) and the effect of the interface between the two gels on heat transfer is negligible. Therefore, a single piece of gel was created in the model with heating and transmission spots added. The physical properties of the materials are summarized in Supplementary Table 2. A time-dependent study was used to acquire the simulation data with a step length of 0.2 s .

### Optical setup

A continuous laser beam (532 nm) was focused on the gel capillary surface with a plano-convex lens with 12.5 cm focal length. The transmitted beam through the channelled PNIPAm was reflected by an angle-adjustable mirror and projected on the PAAm. A linear translation stage was used to change the sample position for tuning the delay distance between the transmission and reflection (heating) spots. A marker was put on the PAAm side to ensure an identical heating position by the reflected beam with respect to the PNIPAm–PAAm interface. A metal block was attached to the PAAm side as the sample holder, as well as an effective heat sink to assist with heat dissipation near the heating spot. The oscillation requires sharp transition of the gel at the transmission spot and time delay between the heating and inhibition processes. Therefore, no oscillation was observed upon non-focused light through PNIPAm, direct photoheating of PNIPAm without delay or using conventional PNIPAm without nanochannels (Supplementary Figs. 4–6, control experiments).

### Fabrication of LCE film actuator

The LCE actuator for the mechano-thermo-mechanical signal transduction was fabricated using a chain extension reaction^41^. Here 0.16 mol of 1,4-bis-[4-(6-acryloyloxyhexyloxy)benzoyloxy]-2-methylbenzene, 0.05 mol of 6-amino-1-hexanol and 0.05 mol of dodecylamine were mixed by magnetic stirring at 85 °C. Then, 2.5 wt% of initiator 2,2-dimethoxy-2-phenylacetophenone was added into the mixture. The mixture was infiltrated into to a cell at 85 °C via capillary force. The cell was prepared by gluing two coated glass substrates, one with a homeotropic alignment layer (JSR OPTMER, 4,000 rpm for 1 min, followed by baking at 100 °C for 10 min and 180 °C for 30 min) and the other with unidirectionally rubbed polyvinyl alcohol (5% water solution, 4,000 rpm for 1 min, baked at 100 °C for 10 min). Then, 50 µm microspheres (Thermo Scientific) were used as spacers to determine the film thickness. The cell was cooled down to 63 °C at 5 °C min^−1^ and kept in the oven for 24 h at 63 °C to allow the aza-Michael addition reaction for oligomerization (Supplementary Fig. 17). Then, the sample was irradiated with UV light (360 nm, 180 mW cm^−2^, 20 min) for polymerization. Finally, the cell was opened by a blade, and strips were cut from the film.

### Fabrication of colour display

Thermochromic dyes (1 wt%) were mixed with polydimethylsiloxane precursor followed by drop casting in a Petri dish mould. The sample was thermally cured in an oven at 80 °C (24 h) to form an elastic film of about 0.5 mm thickness. The polydimethylsiloxane film was cut into 1 mm × 1 mm squares and used as thermochromic stickers. Two types of thermochromic powder pigment from Atlanta Chemical Engineering were used: TP-BP35 (transition from black to pink upon heating above 35 °C) and TP-RC45 (transition from red to white upon heating above 45 °C). Before attaching the thermochromic stickers, a kitchen aluminium foil (10 µm thickness) was cut and stuck onto the top surface of the gel tube, to block the scattered laser from the gel.

### Fabrication of cargo transport system

Three LCE fins (6.00 mm × 1.50 mm × 0.05 mm) were vertically glued on top of a rectangular plastic sheet (4.00 mm × 2.00 mm × 0.10 mm) cut from an Optiazure transparency film. The plastic sheet was placed on top of the capillary tube with LCE fins near the heating spot. Two ends of the plastic sheet were fixed on the capillary surface by double-sided tapes. A U-shaped cargo was made by folding a piece of paper, which was placed on top of the LCE fins. The weight of the paper was 8.5 mg.

### Fabrication of Mimosa- and flytrap-mimic assembly

The gel capillary was fixed on a mechanical stage that can provide a mechanical-trigger-induced displacement (0.3–0.5 mm) along the tube. The stage was connected to a spring, which allows it to return to the original position when the mechanical trigger is removed. For Mimosa-inspired gel–LCE assembly, an LCE strip was placed on top of the heating spot of the capillary. Due to heat-induced softening, the soft LCE spontaneously sticks to the capillary surface. A lightweight fibre is glued on the LCE strip, as an extended rod for better visualization of the bending angle. The LCE strip bends after sensing the heat conducted from the bottom gel capillary. For the flytrap-inspired gel–snapper assembly, a 100-µm-thick plastic strip was first glued with a glass sphere (2 mm diameter) at the centre position. The strip was supported by two objects (3 mm height) on both sides on top of the gel capillary. The sphere was then glued on top of the heating spot of the gel capillary via a liquid crystal ester as a temperature-sensitive adhesive. The plastic strip was pre-bent, and the release of elastic energy was induced by melting of the ester glue, which sharply occurs at 55 °C.

### Optical characterization

Photographs and videos were recorded using a digital camera (Canon 5D Mark III, 100 mm lens), and the camera was equipped with an optical filter with a cut-off wavelength of <500 nm. Thermal images/videos were recorded with an infrared camera (FLIR T420BX, close-up lens with 50 µm resolution). The positions of the LCE strip, sample stage and snapper were tracked by a video analysis software (Kinovea, version 0.9.5).

### Measurement of light transmission

A cover glass slide (0.2 mm thick) was placed between the mirror and gel tube to reflect about 5% of the total power of the light beam transmitted through the channelled PNIPAm gel. The reflected light was measured by a power meter (OP-2 VIS power sensor, Coherent; 1 Hz sampling rate) to determine light transmission (*I*_trans_/*I*_0_) through the gel, where *I*_trans_ is the measured light power during temperature oscillation and *I*_0_ is the initial light power through the PNIPAm gel below its LCST.

## Online content

Any methods, additional references, Nature Portfolio reporting summaries, source data, extended data, supplementary information, acknowledgements, peer review information; details of author contributions and competing interests; and statements of data and code availability are available at 10.1038/s41565-022-01241-x.

### Supplementary information

Supplementary Information Supplementary method for numerical simulation. Supplementary Figs. 1 to 26. Supplementary video captions.

Supplementary Video 1 **Thermal camera movie of stable temperature oscillations in the encapsulated gel**. The oscillation is self-sustained upon a constant light excitation via a delayed feedback loop between two gels in the capillary with an outer dimension of 2.8 × 2.8 mm^2^. Laser beam: 532 nm, 600 mW.

Supplementary Video 2 **Optical camera movie of light-fuelled stable self-oscillation in the encapsulated gel**. The oscillation is self-sustained upon a constant light excitation via a delayed feedback loop between two gels in the capillary with an outer dimension of 2.8 × 2.8 mm^2^. Laser beam: 532 nm, 600 mW. An optical filter is placed in front of the camera to cut off wavelength < 550 nm.

Supplementary Video 3 **Simulation of isothermal surface evolution during stable oscillations**. Simulation power: 340 mW, corresponding to a nominal power of 600 mW in experiments (Fig. 1g&2c). Delay distance *d*: 4.6 mm.

Supplementary Video 4 **Dynamic colour display based on stable oscillation**s. This video shows the colour evolution of thermochromic spots under stable oscillating conditions corresponding to Fig. 4a-e. The laser is switched on at the start of the video. Light power: 700 mW, *d* = 6.1 mm.

Supplementary Video 5 **Cargo transport based on stable oscillations**. This video shows a paper cargo being transported by synchronized LCE fins driven by stable self-oscillation in temperature (Fig. 4i-k). Light power: 700 mW, *d* = 8.1 mm.

Supplementary Video 6 **Mimosa-inspired gel–LCE assembly for single-touch mechanoresponses**. A thermoresponsive soft actuator made of LCE is placed on top of the gel capillary. When a mechanical trigger is applied to the gel, a massive photothermal overshoot is experienced by the heating spot, yielding a thermal bending of the LCE. Laser beam: 532 nm at constant power 700 mW.

Supplementary Video 7 **Flytrap-inspired gel–snapper assembly for frequency-gated mechanoresponses**. A plastic strip is fixed to the gel capillary on top of the heating spot through a temperature sensitive glue. A pre-bending of the strip allows elastic energy to be stored. Upon low frequency triggers, the gel receives limited temperature elevation and shows no response. Upon high frequency triggers, the temperature increases above 55 °C, which melts the glue and triggers a snapping event by the release of elastic energy. Laser beam: 532 nm at constant power 700 mW.

## Data Availability

The data that support the findings of this study are available within the paper and the Supplementary Information. Other relevant data are available from the corresponding authors on reasonable request. Source data are provided with this paper.

## Source data

Source Data Fig. 1 Source data of plots.
Source Data Fig. 2 Source data of plots.
Source Data Fig. 3 Source data of plots.
Source Data Fig. 4 Source data of plots.
Source Data Fig. 5 Source data of plots.
Source Data Extended Data Fig. 1 Source data of plots.
Source Data Extended Data Fig. 2 Source data of plots.
Source Data Extended Data Fig. 3 Source data of plots.
Source Data Extended Data Fig. 4 Source data of plots.
Source Data Extended Data Fig. 5 Source data of plots.
