# Supplementary material for: Feedback-controlled hydrogels with homeostatic oscillations and dissipative signal transduction
Source: Nat Nanotechnol. 2022 Nov 28;17(12):1303–10. doi: 10.1038/s41565-022-01241-x (PMC9747616; doi:10.1038/s41565-022-01241-x)
Supplement: Supplementary file 1 — Supplementary method for numerical simulation. Supplementary Figs. 1 to 26. Supplementary video captions. [file 41565_2022_1241_MOESM1_ESM.pdf]

nature nanotechnology

Article

https://doi.org/10.1038/s41565-022-01241-x

Feedback-controlled hydrogels with homeostatic oscillations and dissipative signal transduction

In the format provided by the authors and unedited

Table of Contents

1. Supplementary method for numerical simulation 1

2. Supplementary figures 3

3. Supplementary video captions 29

1. Supplementary method for numerical simulation

The simulation of the oscillator was carried out in COMSOL Multiphysics 5.5. The geometry parameters used to set up the 3D model are summarized in Supplementary Table 1 and Supplementary Fig. 10. It is assumed that the two gels possess the same physical parameters (heat capacity, thermal conductivity, and density) and the effect of the interface between the two gels on heat transfer is negligible. Therefore, a single piece of gel was created in the model. The physical properties of the materials are summarized in Supplementary Table 2.

Supplementary Table 1. Geometries of components in the simulation model

Components	Length (mm)	Width (mm)	Height (mm)	Diameter (mm)
Glass capillary	40	2.0 (inner) × 2.8 (outer)	2.0 (inner) × 2.8 (outer)	n.a.
Gel	40	2.0	2.0	n.a.
Metal block	20	20	20	n.a.
Heating spot	n.a.	n.a.	1.2	1.2
Transmission spot	n.a.	n.a.	2.0	0.2

Supplementary Table 2. Physical properties of the materials in the model

Material	Heat capacity (J kg^{-1} K^{-1})	Density (kg m^{-3})	Thermal conductivity (W m^{-1} K^{-1})
Glass	754	2230	1.13
Gel	4035.840 + 0.492 × *T**	1030	0.58
Metal	475	7850	50

*: *T* is the absolute temperature in Kelvin below 373K. Above 373K a constant heat capacity is used as at 373K.

To simulate the physical processes, a heat transfer module (Heat Transfer in Solids and Fluids) has been used. An artificial cylindrical heat source is added to the heating spot to represent the photothermal heating caused by the laser. The power of the heat source is dependent on the average temperature at the transmission spot via an exponential Equation S1:

$$P = P_0 \times \frac{A}{1+e^{\frac{T_1 - T_{LCST}}{0.2}}} \quad \text{(S1)}$$

where P is the heating power on the heating spot, P_0 the incident laser power, A a constant between 1 and 0.75, T_1 the average temperature of the transmission spot, and T_{LCST} the LCST of PNIPAm (35.9 °C). The exponential term $\frac{A}{1+e^{\frac{T_1 - T_{LCST}}{0.2}}}$ is utilized to describe the phase transition of the channelled PNIPAm, which is plotted in Supplementary Fig. 1 with $A = 1$. The value of A is set at 1 at the start of oscillation, which is then adjusted to a value between 0.75 and 1 after the first cycle of oscillation. This is to account for the fact that the laser intensity in the experiments does not fully return to 100% after the first cycle, as shown in Extended Data Fig. 1. The temperature on the top surface of the metal block is set as a constant at room temperature. The heat transfer between the glass capillary and the metal block is treated as non-ideal, and a *Constriction conductance with interstitial gas* model is used to simulate the thermal resistance. The contact pressure is 10 N m^{-2} and the surface roughness (12 – 20 um) is a fitting parameter for each set of data. *Time dependent* study is used with a step length of 0.2 s.

The nominal laser power, i.e., the experimental laser power measured at the laser source, has been used throughout the main text. For simulation, the laser power P_0 at the heating spot is a fitting parameter for each data set due to the loss of laser intensity along the optical path (reflection, scattering at interfaces etc.), which varies depending on the samples and the setup. For the power-dependent series (Fig. 2h), the ratio between different P_0 is the same as the nominal power. The nominal power and P_0 used in this study have been summarized in Supplementary Table 3.

Supplementary Table 3. Nominal laser power and simulation power P_0

Dataset	Nominal laser power (mW)	Simulation power P_0 (mW)
Fig. 2c	600	340
Fig. 2g	600	310
Fig. 2h	460	176
Fig. 2h	600	230
Fig. 2h	675	258
Fig. 2h	700	267

2. Supplementary figures

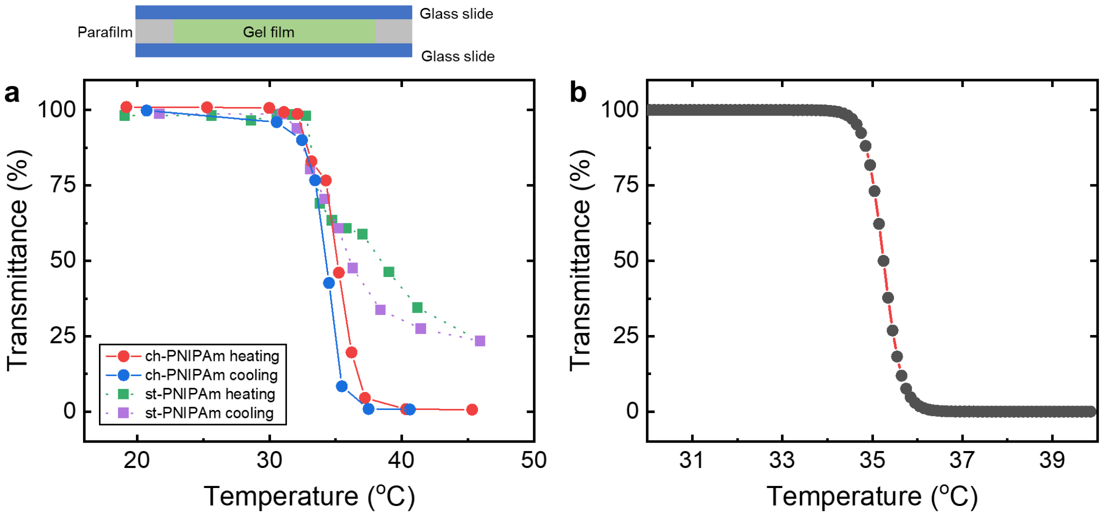

Supplementary Fig. 1 | Phase transition of PNIPAm. a, Measured transition curves (transmission at 532 nm) for channelled (ch-) and standard (st-) PNIPAm upon heating and cooling. Top panel shows setup used for measuring the gel samples, with a fixed thickness of 0.5 mm. **b,** Exponential curve used in simulating the phase transition of PNIPAm.

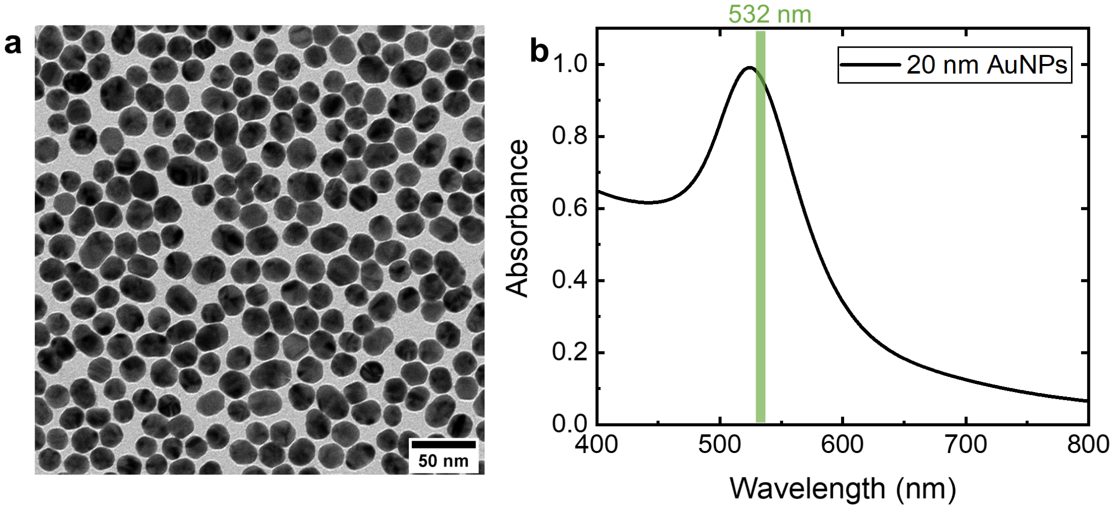

Supplementary Fig. 2 | Characterization of AuNPs. a, TEM image of the AuNPs. **b,** UV-Vis spectrum of the AuNPs with 532 nm marked by the green line. Note that the concentration of AuNPs used in this spectrum is half of that used in the gel oscillator at the same optical path length (2 mm). The absorbance of the oscillator at 532 nm is 2.

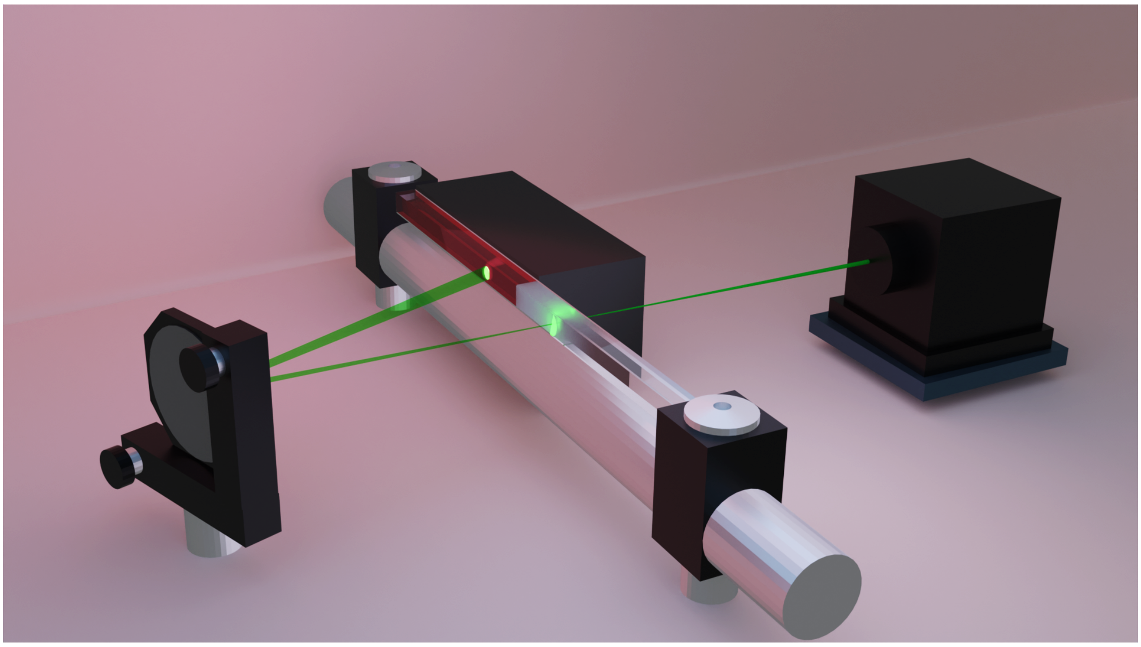

Supplementary Fig. 3 | The optical set-up of the gel oscillator. A continuous laser beam (532 nm) is focused by a Plano-convex lens (12.5 cm focal length) onto the PNIPAm gel (white), while the transmitted light is reflected to the PAAm/AuNP gel via a mirror. The capillary is clamped on both sides by two metal stages, while the PAAm part of the capillary is attached to a vertically orientated metal block for heat dissipation.

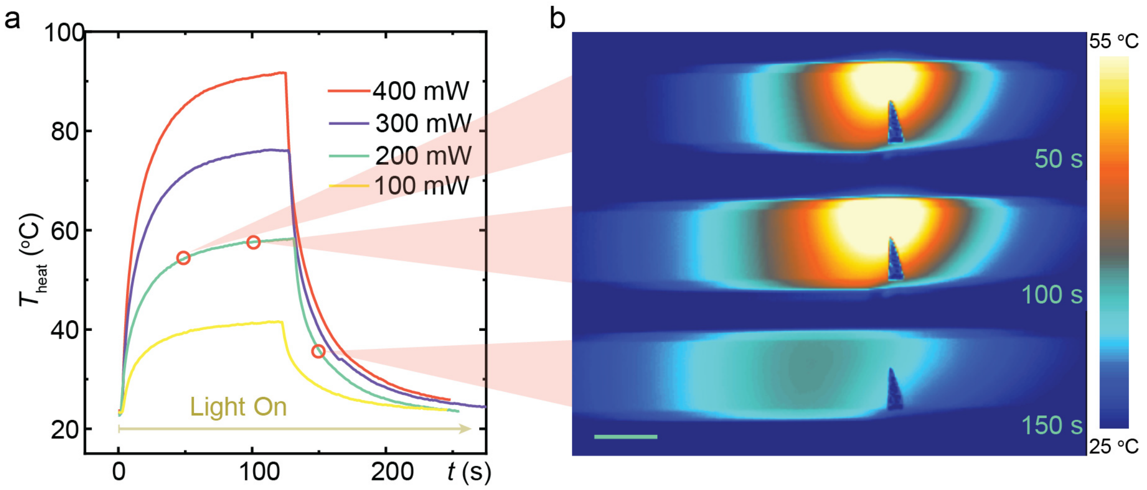

Supplementary Fig. 4 | Gel response upon non-focused laser excitation. a, The temperature curve, indicating significant damping upon laser transmission through PNIPAm with a spot size of 2 mm. **b**, Thermal camera images of the gel capillary at different damping stages. Scale bar: 2 mm.

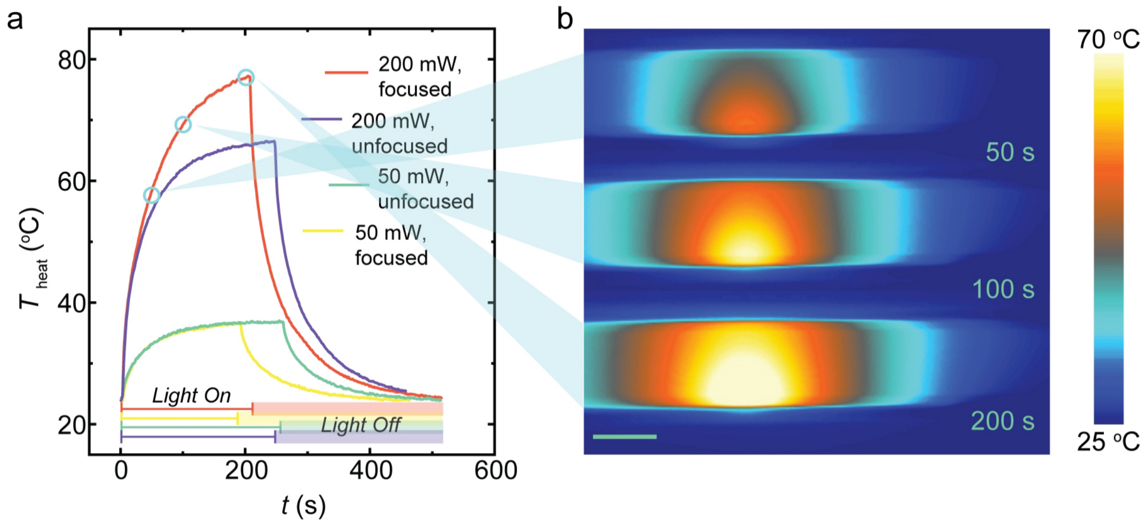

Supplementary Fig. 5 | Gel response to direct laser excitation with zero delay. a, Temperature variation curve, showing rapid saturation in temperature elevation upon different power excitations. **b**, Thermal camera images of the gel capillary at 50 s, 100 s, 200 s, upon 200 mW focused light irradiation. Scale bar: 2 mm.

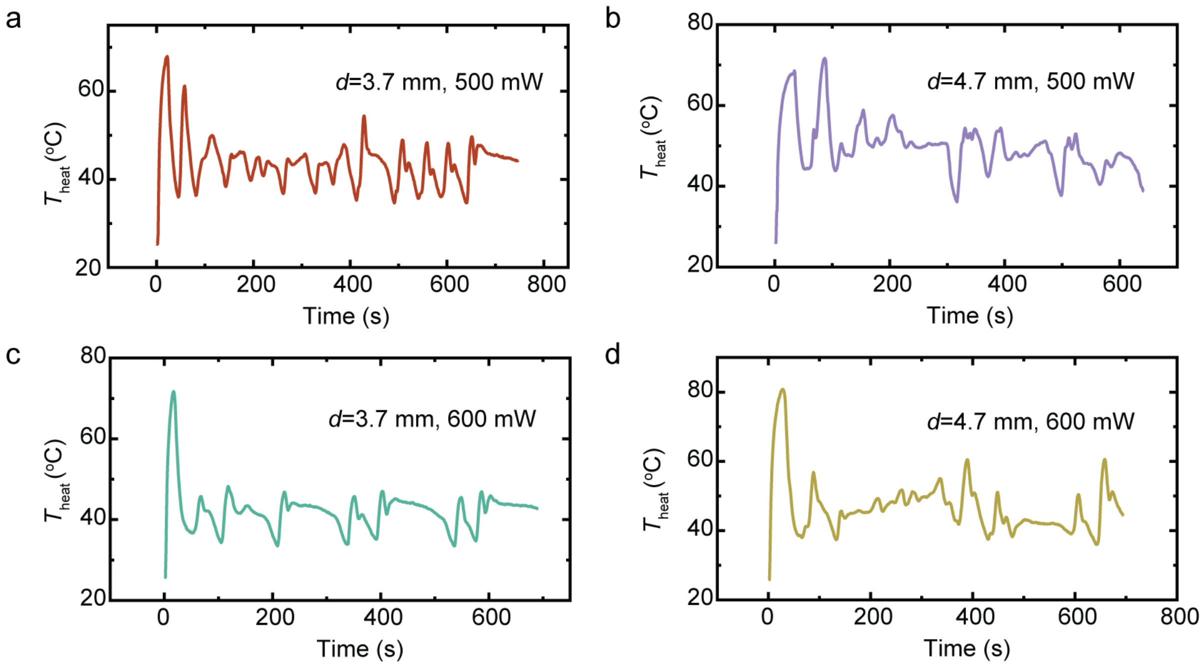

Supplementary Fig. 6 | The light response of feedback system using conventional PNIPAm hydrogels without nano-channels. Different delay distance *d* and excitation power are selected for the measurements, as indicated in each figure. The same optical set-up has been used as with nano-channelled PNIPAm, shown in Supplementary Fig. 3.

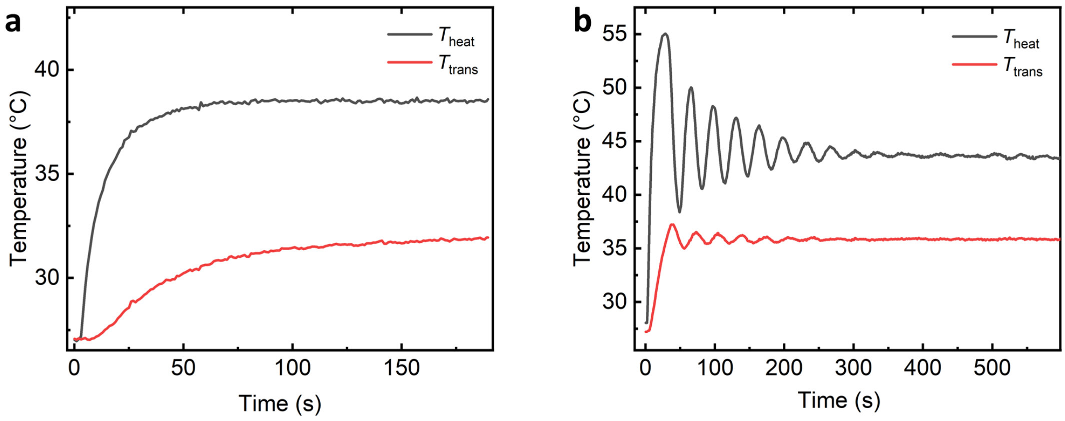

Supplementary Fig. 7 | Representative examples of damping and insufficient laser power. a, Temperature evolution under insufficient laser power (100 mW), d = 4.1 mm. **b,** Damping of oscillations at 300 mW, d = 4.1 mm.

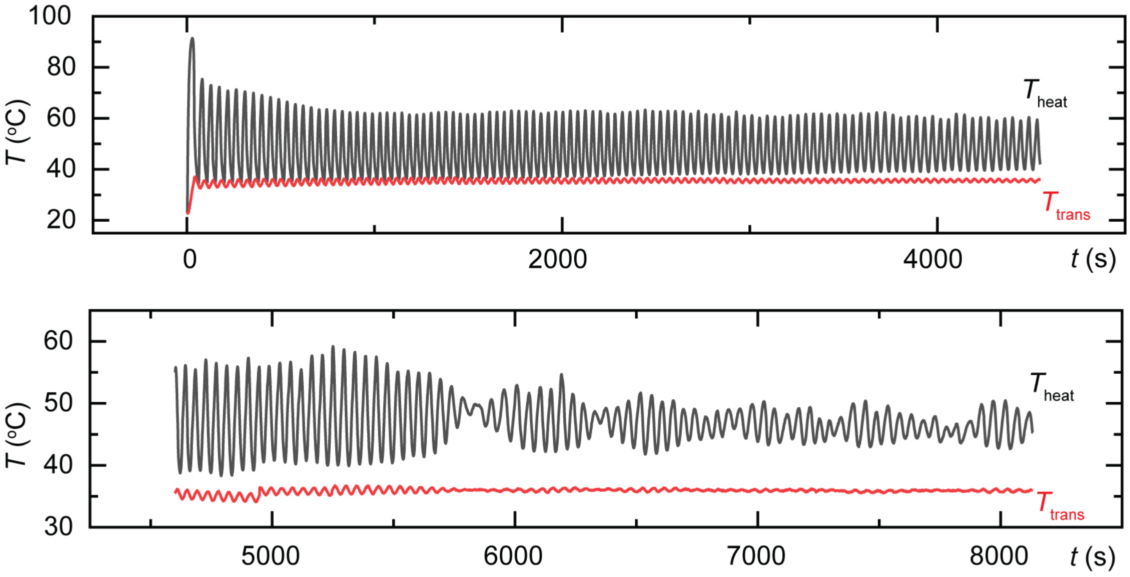

Supplementary Fig. 8 | Stable self-oscillations. Laser: 600 mW at 532 nm. Delay distance: 4.6 mm.

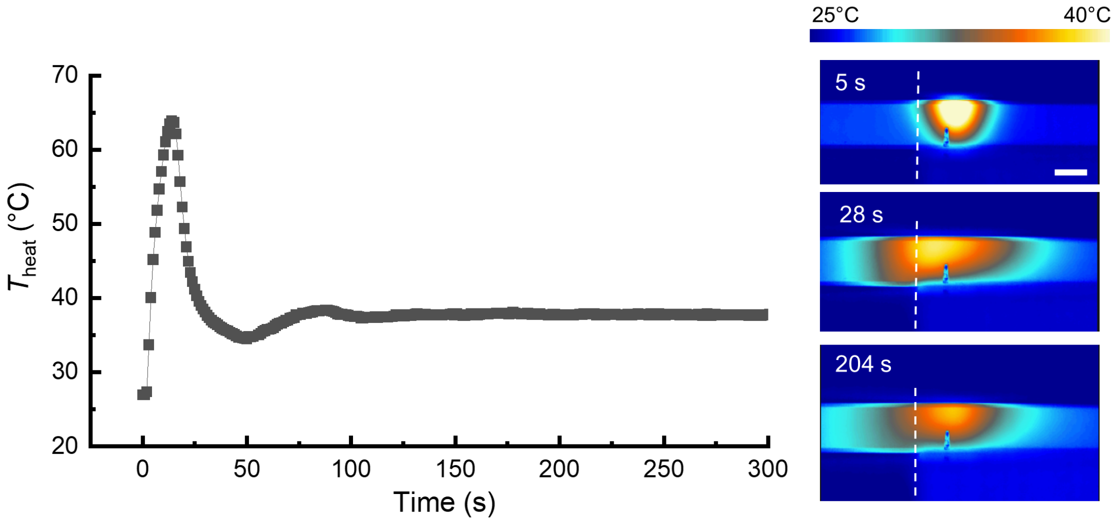

Supplementary Fig. 9 | Scattering-induced heating at gel-gel interface at short *d*. The dashed lines indicate the interface of the two gels. Note that at 28 s, the hot spot shifted towards the interface between the gels, indicating partial contribution to heating at the interface. This leads to quick damping of the oscillation. d = 2.8 mm; Power = 800 mW. Scale bar: 2 mm.

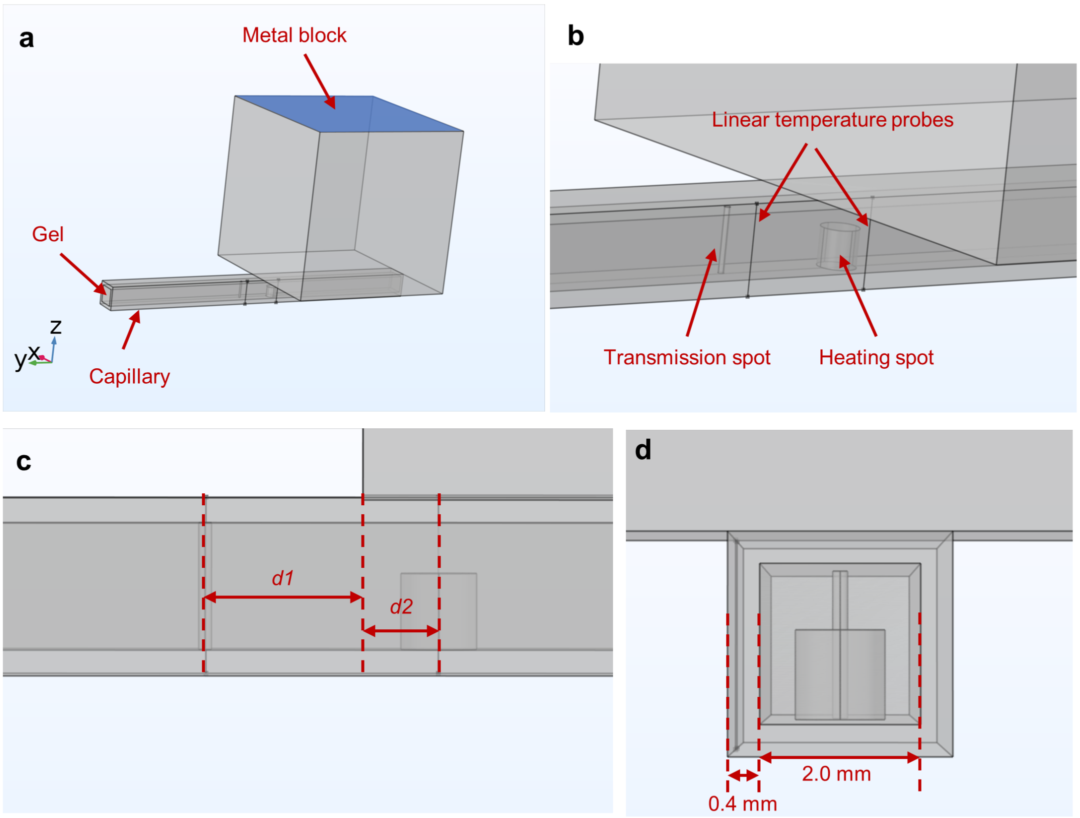

Supplementary Fig. 10 | Dimensions of the simulation model. **a,** three components of the model indicated by the arrows. The blue-highlighted surface is fixed at room temperature. **b,** Zoom-in of the model showing the transmission spot, heating spot, and the linear temperature probes. **c,** Distance between the transmission spot and the boundary of the metal block (*d1*) and distance between the heating spot and the boundary of the metal block (*d2*). **d,** Dimension of the cross-section of the gel and the capillary.

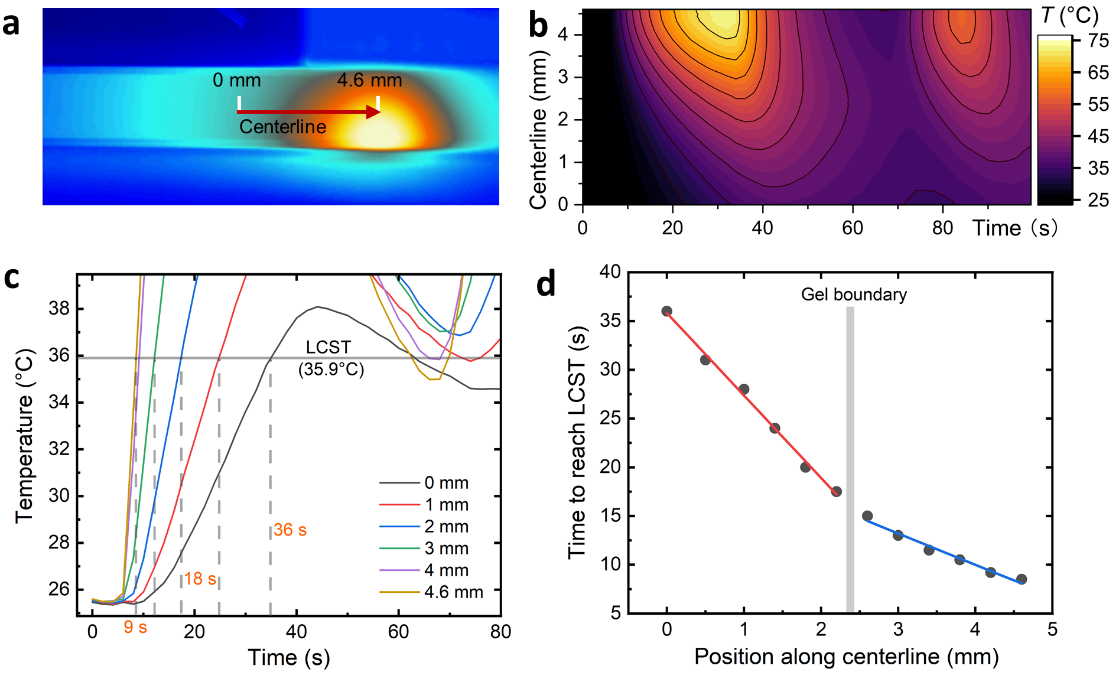

Supplementary Fig. 11 | Temperature evolution along the centerline of gel oscillator. a, Illustration of the centerline between transmission spot (0 mm) and heating spot (4.6 mm). **b,** Colour map of temperature evolution along the centerline. **c,** Temperature change at different positions along the centerline at the onset of irradiation. Irradiation starts at t = 4 s. **d,** Time to reach LCST (35.9°C) at different positions along the centerline from (c), and the linear fits separated by the boundary between PNIPAm and PAAm gels. The two slopes are due to the different heat dissipation conditions of the two gels. d = 4.6 mm, power = 600 mW.

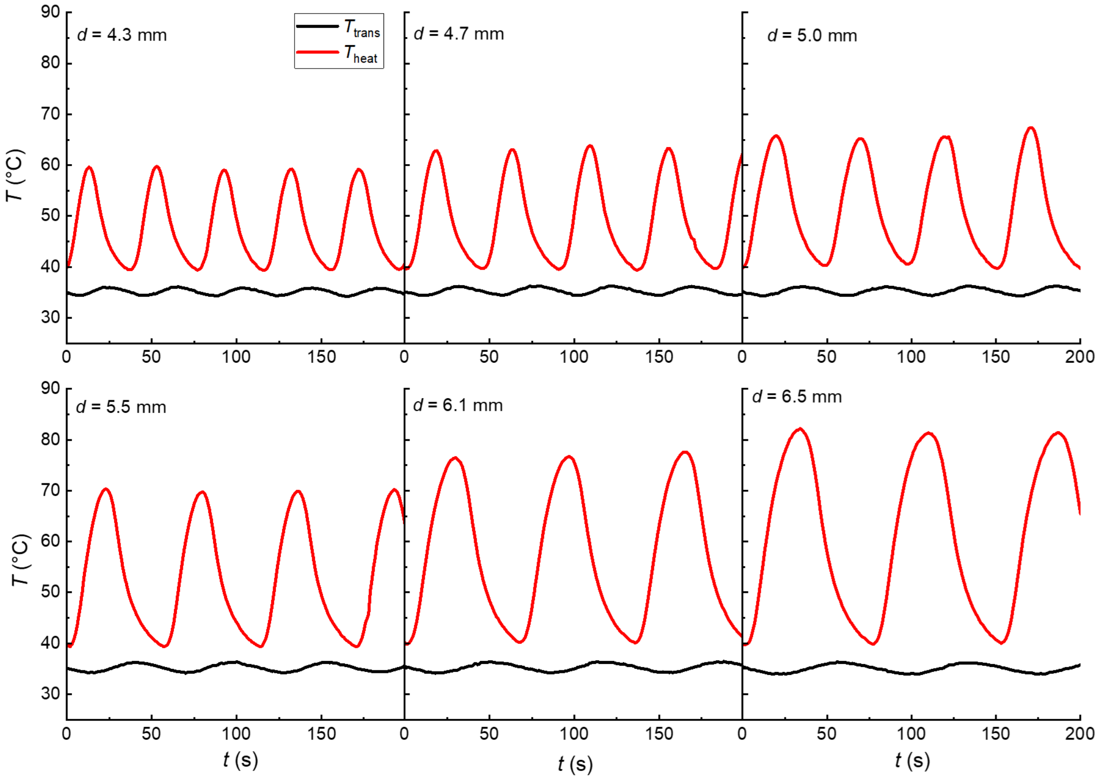

Supplementary Fig. 12 | Experimental results of distance-dependent oscillations. The delay distance d is marked at each panel. Power = 600 mW.

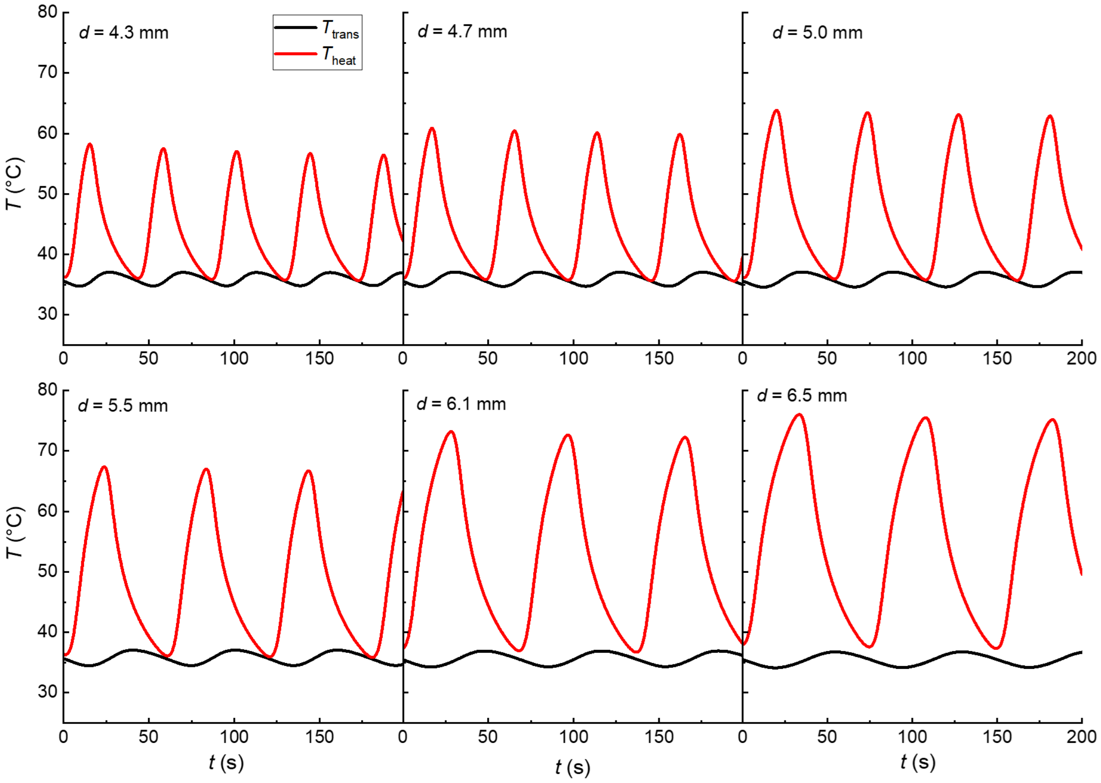

Supplementary Fig. 13 | Simulation results of distance dependent oscillations. The delay distance *d* is marked on each panel. Nominal power = 600 mW.

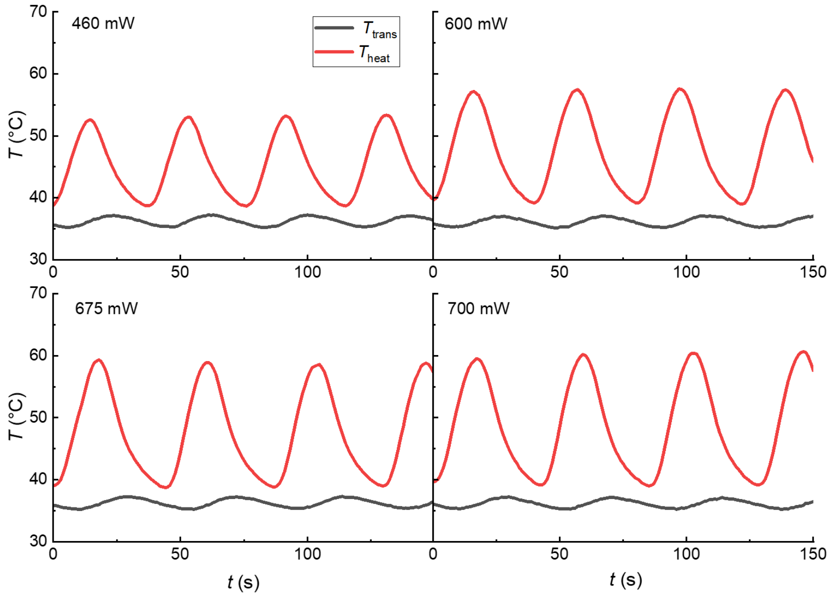

Supplementary Fig. 14 | Experimental results of power-dependent oscillations. The laser power used is marked on each panel. $d = 4.1$ mm

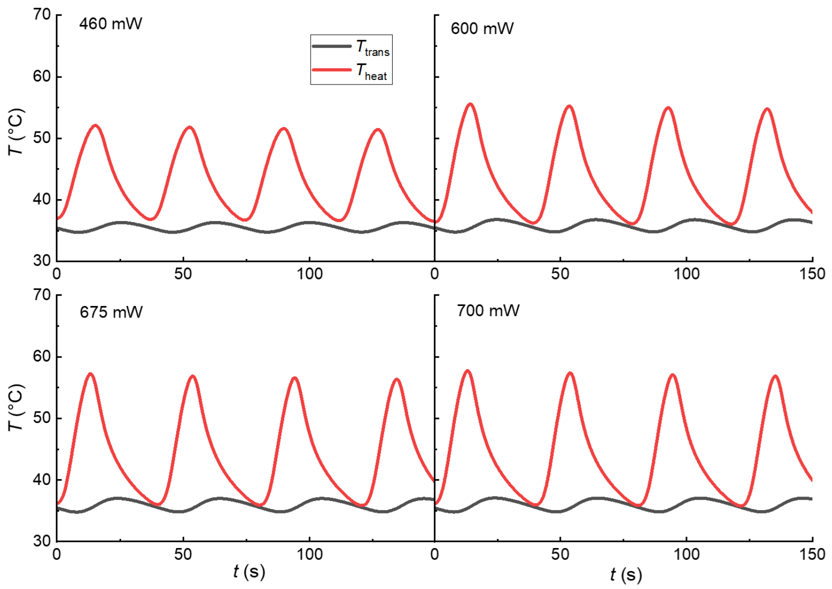

Supplementary Fig. 15 | Simulation results of power-dependent oscillations. The nominal laser power used is marked on each panel. d = 4.1 mm.

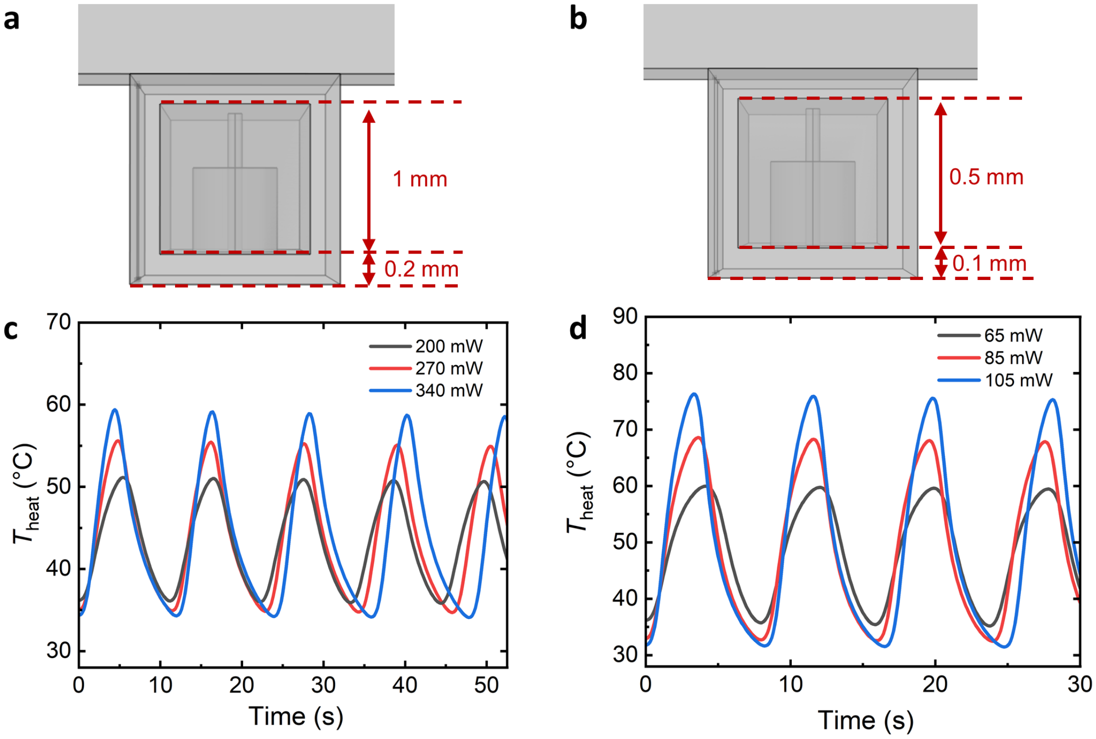

Supplementary Fig. 16 | Simulation of size-dependent oscillation of T_{heat}. a, Illustration of the simulation model with half the size of the original oscillator. **b,** Illustration of the simulation model with one fourth the size of the original oscillator. **c,** The oscillation of T_{heat} in model shown in (a). **d,** The oscillation of T_{heat} in model shown in (b). Nominal powers are indicated in (c) and (d), which is 2 times of the simulation power similar to the other simulations. The other dimensions of the oscillator setup have been reduced by the same factors, respectively. The physical parameters are the same as used for Fig. 2c. The delay distance *d* is fixed at 2 mm for both cases.

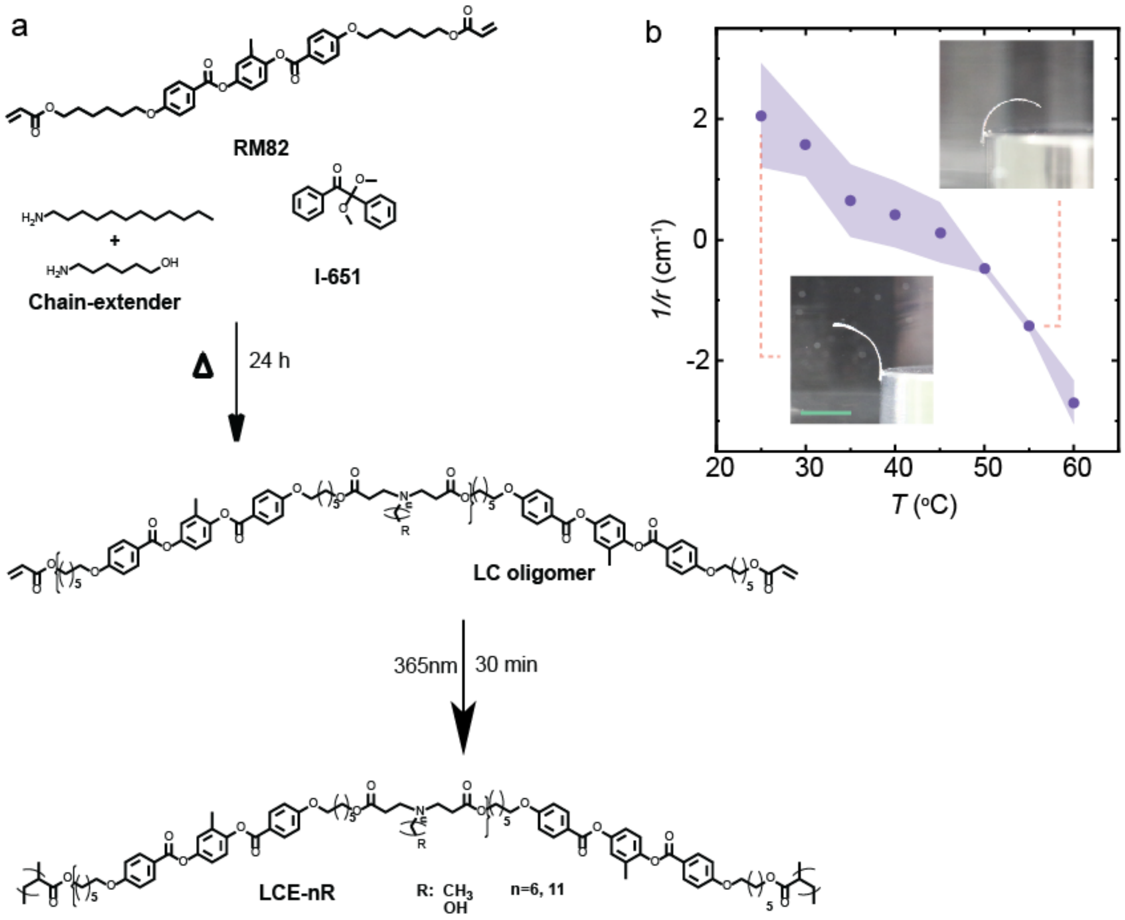

Supplementary Fig. 17 | Thermoresponsive liquid crystal elastomer (LCE) actuator. a, Chemicals and synthesis steps for fabricating the LCE. **b**, Change of the LCE strip curvature upon heating. The curvature is defined as 1/r, where r is the bending radius. The error bars indicate standard deviation for n = 3 measurements. Scale bar: 5 mm.

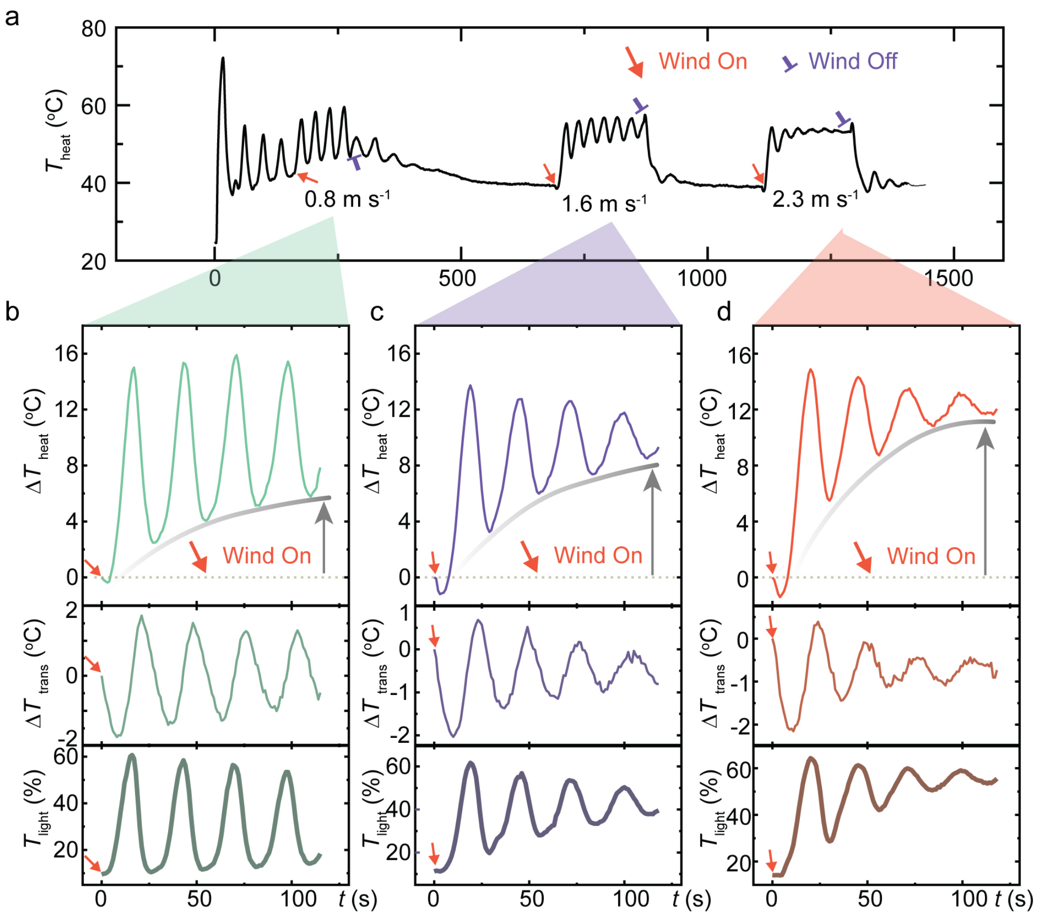

Supplementary Fig. 18 | Response to different wind speed. a, Change of the oscillation temperature by reacting to wind gust of different velocities. Change of temperature at the heating spot, transmission spot and light transmittance (T_{light}) upon 0.8 m s^{-1} (**b**), 1.6 m s^{-1} (**c**), 2.3 m s^{-1} (**d**) wind disturbances. Laser power: 600 mW.

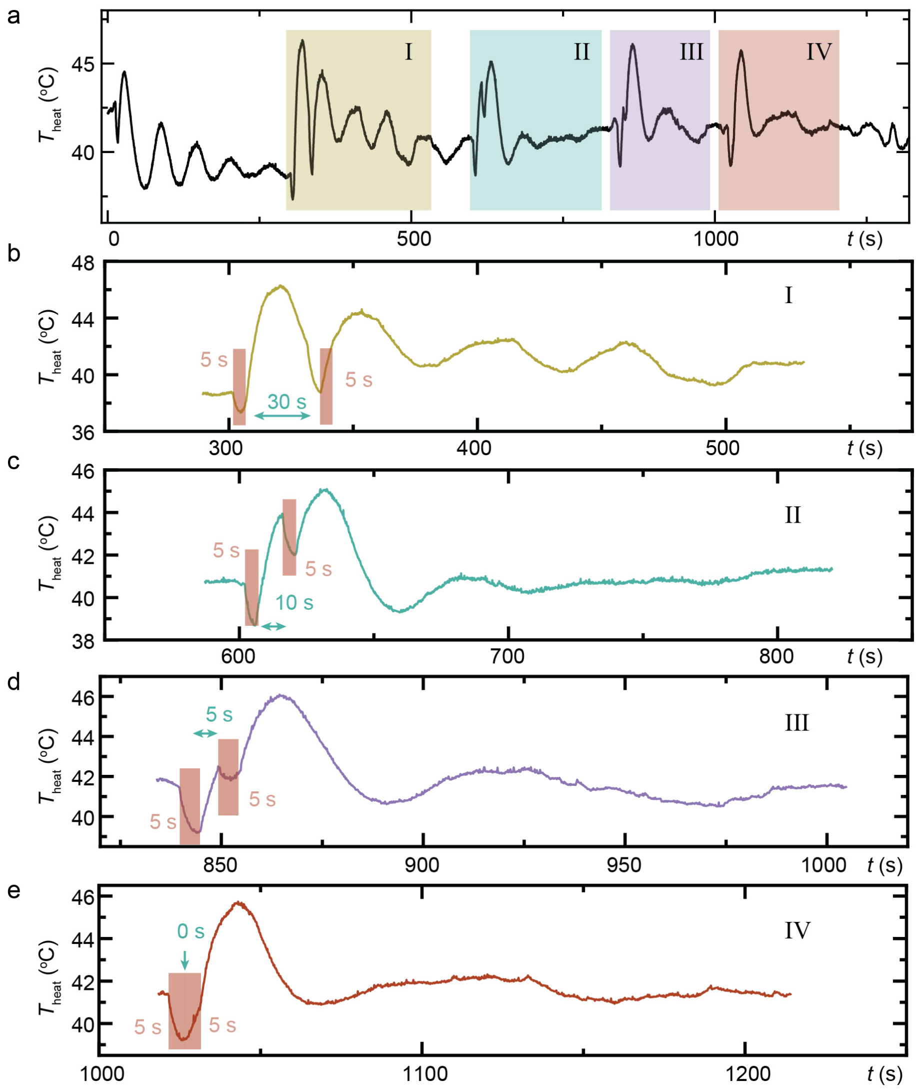

Supplementary Fig. 19 | Response to different wind gaps. a, Change of the oscillating temperature by reacting to two wind gusts with different time intervals between them. Change of temperature at the heating spot upon two wind gusts with 30s (**b**), 10 s (**c**), 5s (**d**) and 0 s (**e**) gaps. Wind gust: 1.6 m s^{-1}, 5 s. Laser power: 800 mW.

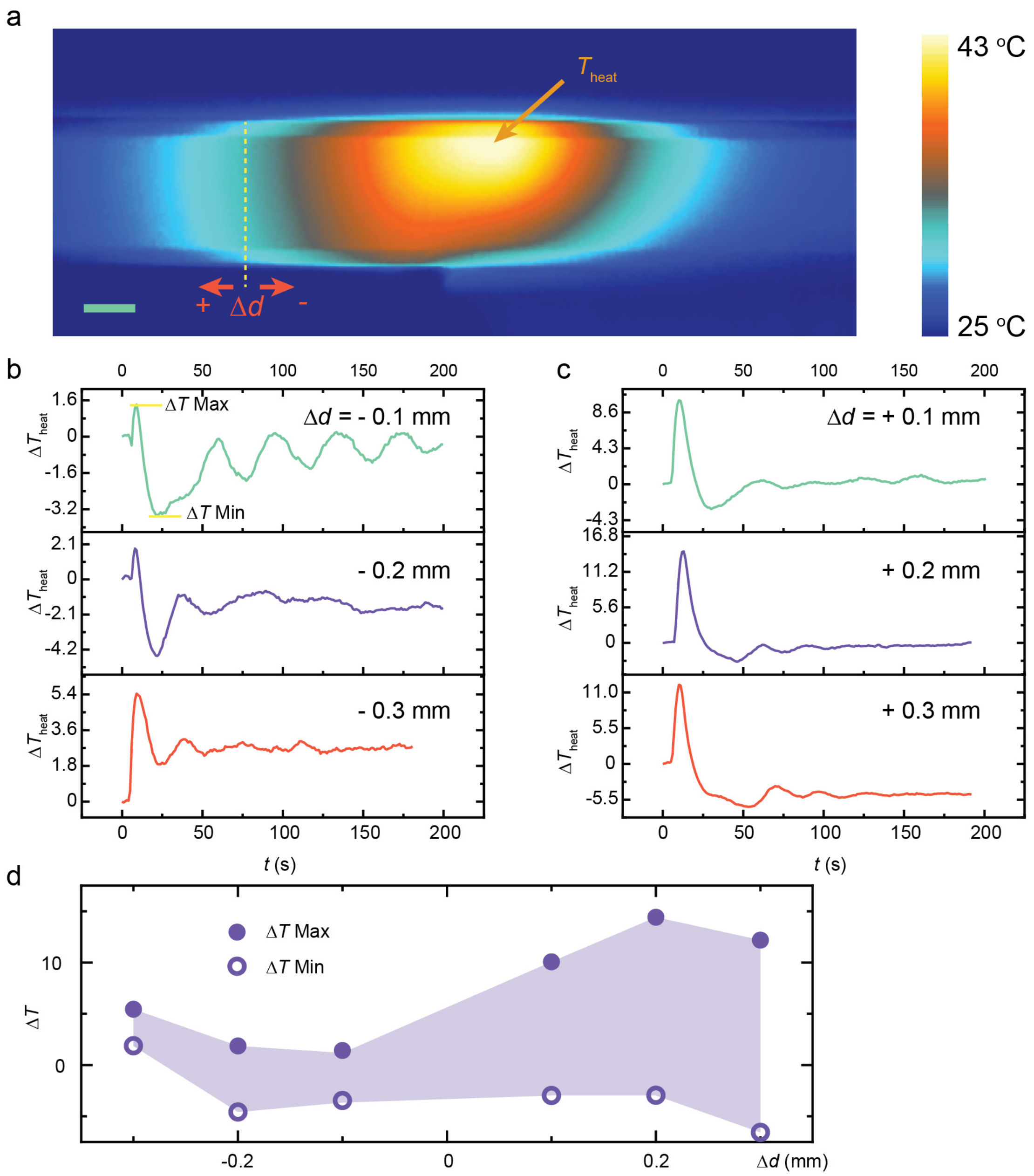

Supplementary Fig. 20 | Response to the change of delay distance. a, Thermal camera image of the gel capillary, and indication of the tuning of the delay distance by changing the input laser position (dashed line). Scale bar: 1 mm. Change of the oscillation temperature by slightly reducing (**b**) and increasing (**c**) the delay distance. **d**, The maximal and minimal temperatures recorded after each disturbance.

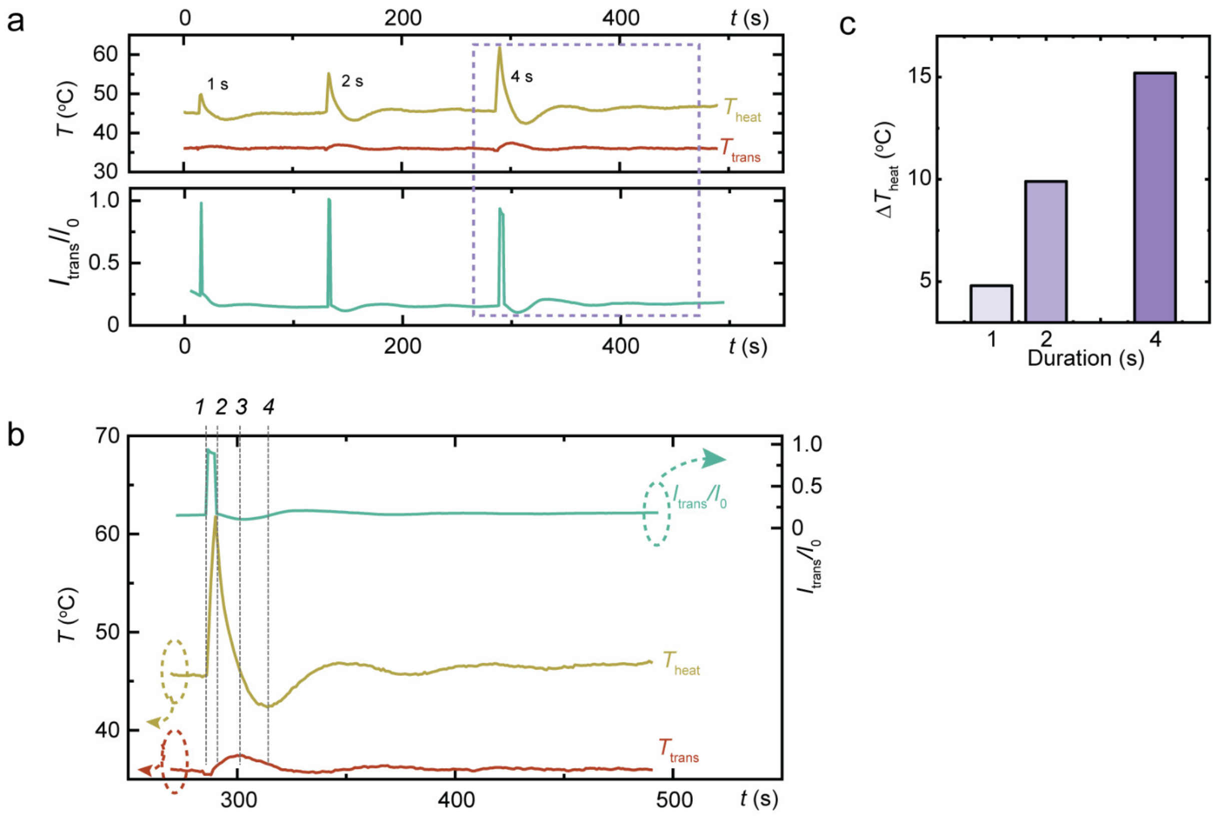

Supplementary Fig. 21 | Response to different duration of a mechanical trigger. a, Temperature and light transmission change after mechanical stimuli with 1, 2, 4 s duration. **b**. Zoom-in data of the dashed frame shown in (a), indicating the correlation between light transmission through the gel (I_{trans}/I_0) and T_{heat} (temperature of heating spot), and time-delay between the T_{heat} and T_{trans} (temperature of transmitted spot). **c**, The elevated temperature upon triggers with different duration. Laser power: 600 mW, delay distance: 5.4 mm. I_{trans} is the detected light power through the PNIPAm gel, and I_0 is the initial light power through the PNIPAm gel below LCST.

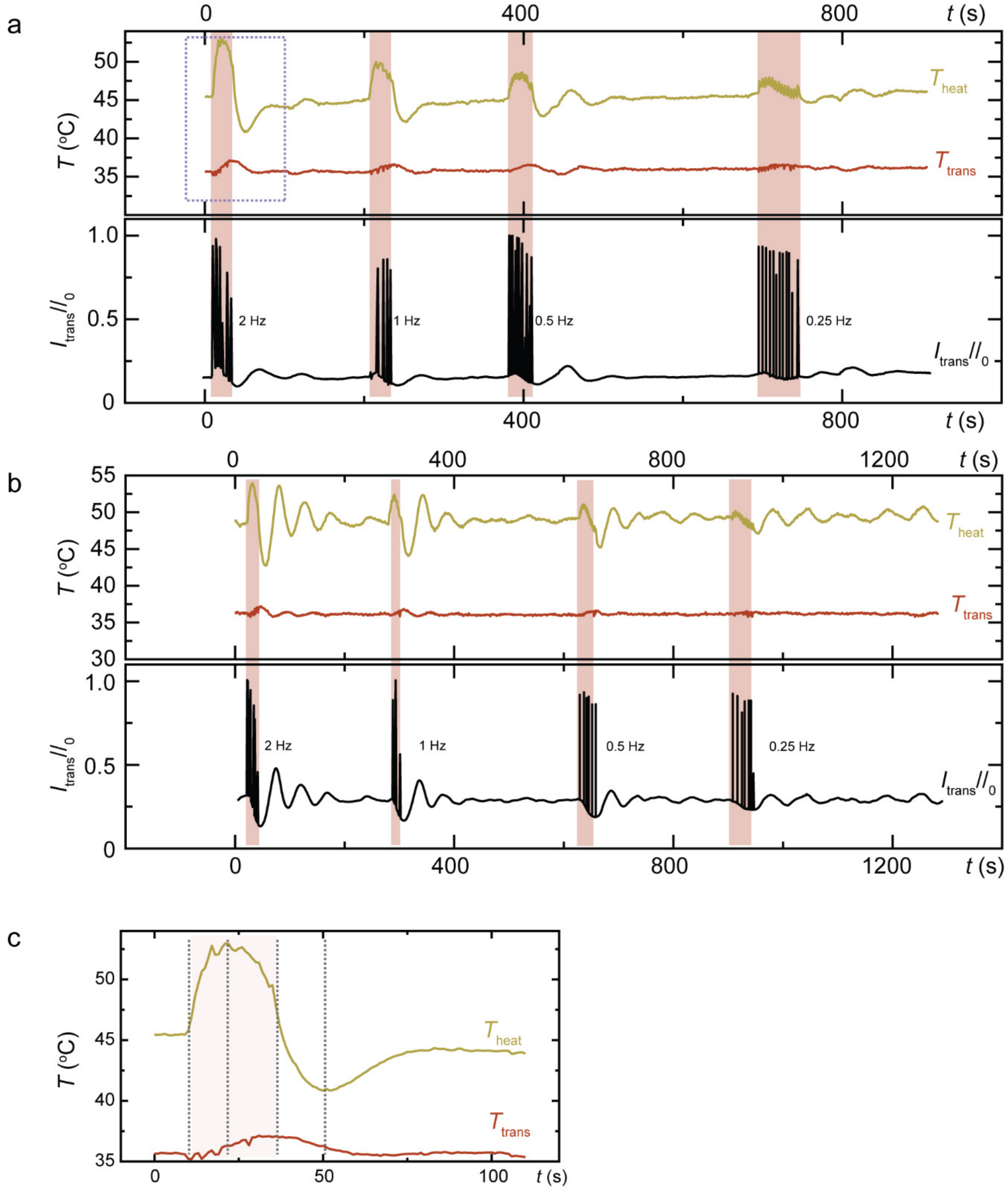

Supplementary Fig. 22 | Response to frequency of mechanical triggers. Temperature and light transmission change after mechanical triggers applied at 2, 1, 0.5, and 0.25 Hz frequency at (**a**) power 600 mW, delay distance 5.1 mm and (**b**) power 700 mW, delay distance 4.7 mm. **c**, Zoom-in data of the dashed frame shown in (a), indicating the time-delay between the T_{heat} and T_{trans}. I_{trans} is the measured light power through the PNIPAm gel, and I_0 is the initial light power through the PNIPAm gel below LCST.

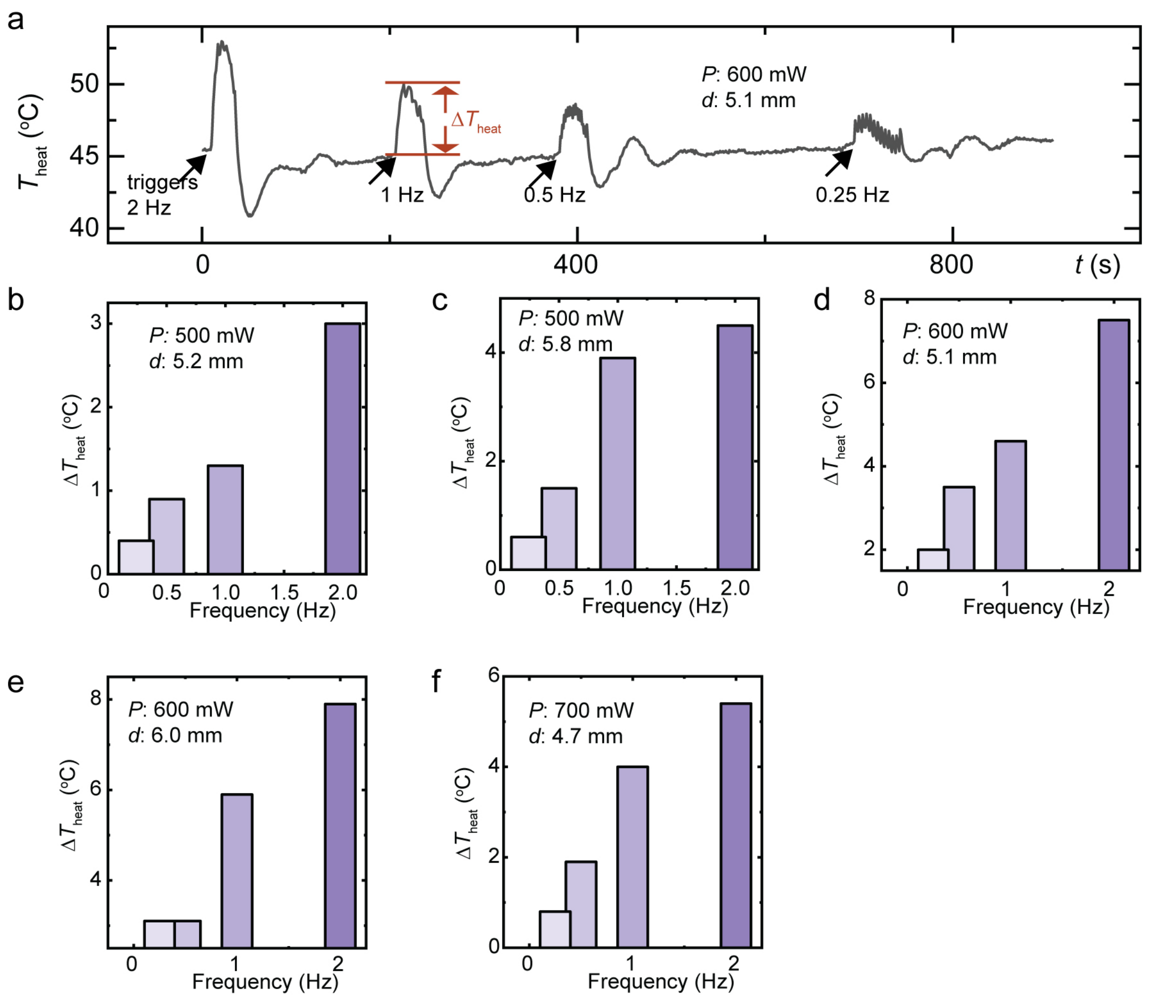

Supplementary Fig. 23 | Frequency dependent temperature elevation. a, Temperature change after mechanical triggers applied at 2, 1, 0.5, and 0.25 Hz frequency. Power *P*: 600 mW, *d*: 5.1 mm. Maximal temperature elevation upon different frequent triggers at (**b**) *P*: 500 mW, *d*: 5.2 mm, (**c**) *P*: 500 mW, *d*: 5.8 mm, (**d**) *P*: 600 mW, *d*: 5.1 mm, (**e**) *P*: 600 mW, *d*: 6.0 mm and (**f**) *P*: 700 mW, *d*: 4.7 mm.

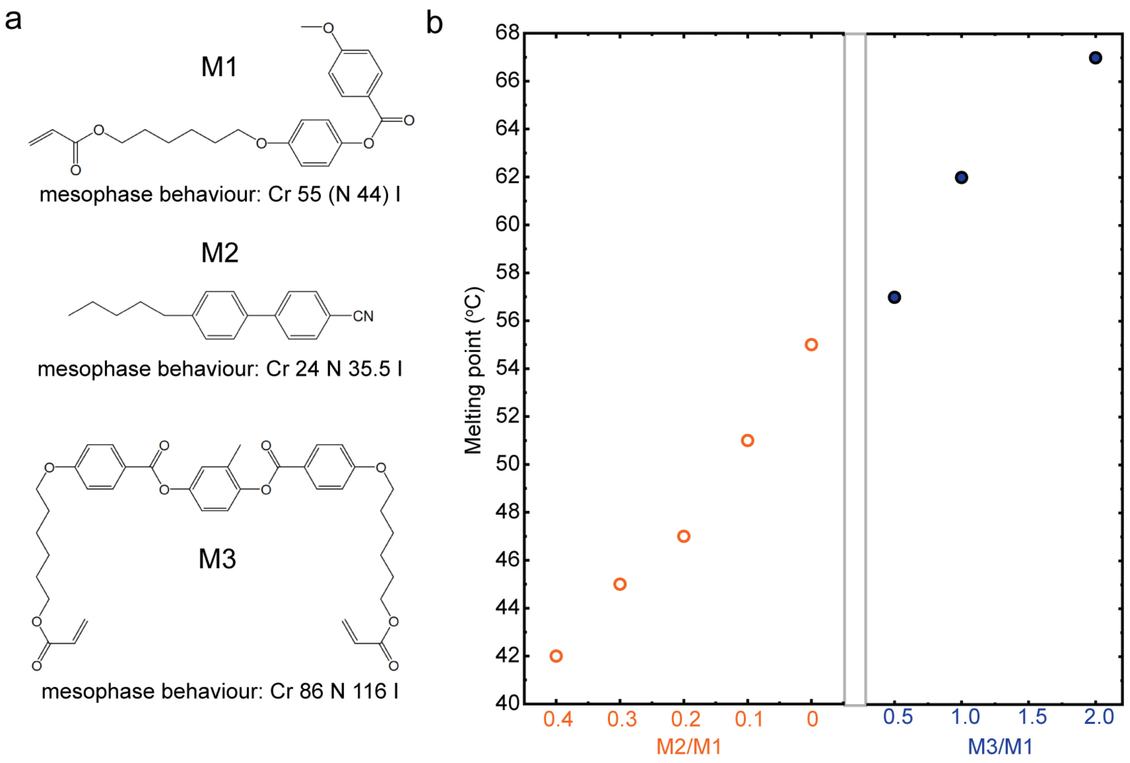

Supplementary Fig. 24 | Melting point tunability. a, Chemical structures and mesophase behaviours of three liquid crystal molecules in use. **b,** Solid-to-liquid transition temperature upon heating of liquid crystalline mixtures with different chemical compositions.

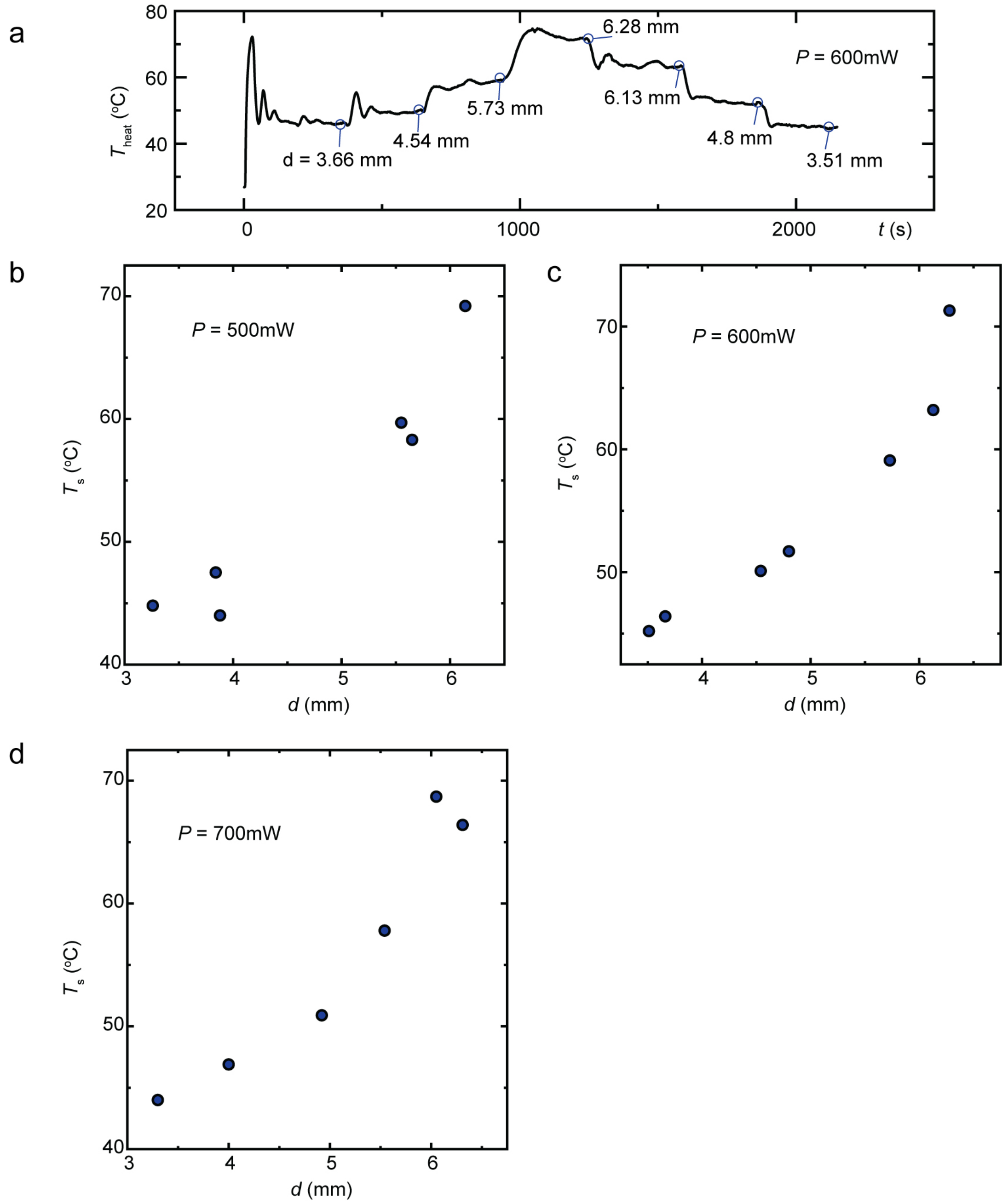

Supplementary Fig. 25 | Tuning of the steady-state temperature. a, Tuning of the steady state temperature T_s by adjusting the delay distance d. Laser power: 600 mW. The obtained T_s at different delay distance under excitation of (**b**) 500 mW, (**c**) 600 mW and (**d**) 700 mW laser power.

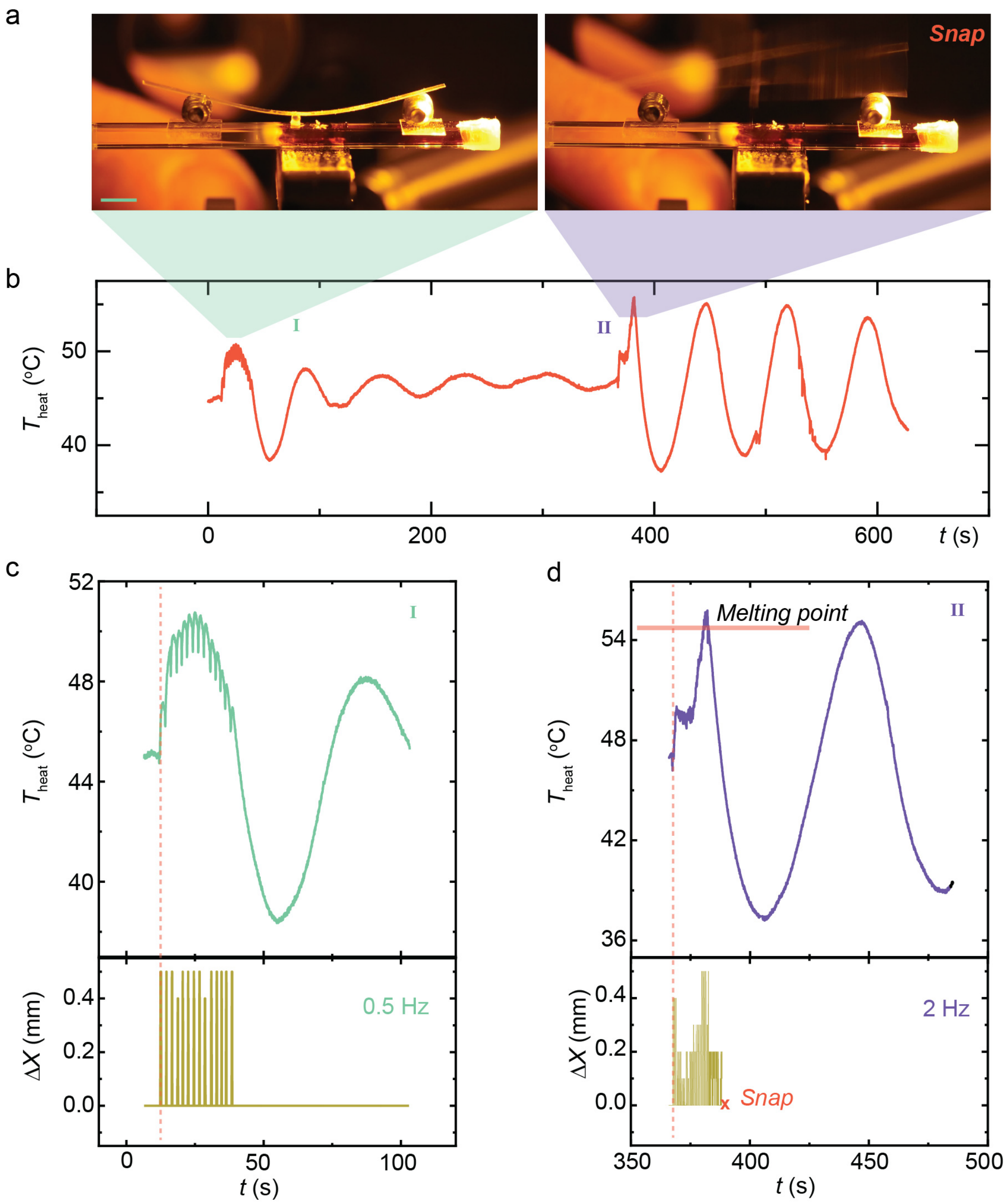

Supplementary Fig. 26 | Frequency-gated snapping example. a, Photographs of the gel-snapper assembly, showing no response upon low-frequency mechanical triggering and snapping upon high-frequency mechanical triggering. Scale bar: 5 mm. **b**, Temperature change during the mechanical triggering. **c**, Zoom-in of temperature change upon 0.5 Hz triggering, and the related sample position change D*x*. **d**, Zoom-in of temperature change upon 2 Hz triggering, and the related sample position change D*x*. Laser power: 700 mW.

3. Supplementary video captions

Supplementary video 1. Thermal camera movie of stable temperature oscillations in the encapsulated gel. The oscillation is self-sustained upon a constant light excitation via a delayed feedback loop between two gels in the capillary with an outer dimension of 2.8 × 2.8 mm^2. Laser beam: 532 nm, 600 mW.

Supplementary video 2. Optical camera movie of light fueled stable self-oscillation in the encapsulated gel. The oscillation is self-sustained upon a constant light excitation via a delayed feedback loop between two gels in the capillary with an outer dimension of 2.8 × 2.8 mm^2. Laser beam: 532 nm, 600 mW. An optical filter is placed in front of the camera to cut off wavelength < 550 nm.

Supplementary video 3. Simulation of isothermal surface evolution during stable oscillations. Simulation power: 340 mW, corresponding to a nominal power of 600 mW in experiments (Fig. 1g&2c). Delay distance *d*: 4.6 mm.

Supplementary video 4. Dynamic colour display based on stable oscillation. This video shows the colour evolution of thermochromic spots under stable oscillating conditions corresponding to Fig. 4a-e. The laser is switched on at the start of the video. Light power: 700 mW, d = 6.1 mm.

Supplementary video 5. Cargo transport based on stable oscillation. This video shows a paper cargo being transported by synchronized LCE fins driven by stable self-oscillation in temperature (Fig. 4i-k). Light power: 700 mW, d = 8.1 mm.

Supplementary video 6. Mimosa-inspired gel-LCE assembly for single-touch mechanoresponses. A thermoresponsive soft actuator made of liquid crystal elastomer (LCE) is placed on top of the gel capillary. When a mechanical trigger is applied to the gel, a massive photothermal overshoot is experienced by the heating spot, yielding a thermal bending of the LCE. Laser beam: 532 nm at constant power 700 mW.

Supplementary video 7. Flytrap-inspired gel-snapper assembly for frequency-gated mechanoresponses. A plastic strip is fixed to the gel capillary on top of the heating spot through a temperature sensitive glue. A pre-bending of the strip allows elastic energy to be stored. Upon low frequency triggers, the gel receives limited temperature elevation and shows no response. Upon high frequency triggers, the temperature increases above 55 ºC, which melts the glue and triggers a snapping event by the release of elastic energy. Laser beam: 532 nm at constant power 700 mW.
